# Potential Vaccine or Antimicrobial Reagents: Simple Systems for Producing Lambda Display Particles (LDP) and Sheathed Lambda DNA Vaccine Particles (LDNAP)

**DOI:** 10.3390/v17111406

**Published:** 2025-10-22

**Authors:** Sidney Hayes

**Affiliations:** Department of Biochemistry, Microbiology and Immunology, College of Medicine, University of Saskatchewan, Saskatoon, SK S7N 5E5, Canada; sidney.hayes@usask.ca or sjh092iii@outlook.com

**Keywords:** vaccine and antimicrobial agents, system for preparing phage display particles and phage-encapsulated DNA vaccines, simultaneous-multiple single epitope vaccine preparation strategy

## Abstract

The focus of this study was to explore phage display systems employing bacteriophage lambda (λ) gene fusions to its capsid decoration protein gpD as reagent tools for tackling disease. The biological activity of gpD-fusions was examined by testing for the retained antimicrobial toxicity of cathelicidins or defensins fused to gpD. Our previous finding that only COOH fusions of either cathelicidins or defensins to gpD were toxigenic was expanded to show that only the reduced form of fused defensin antimicrobial polypeptides was found to be toxigenic. Compared in review are gene-fusion lytic display systems (where the fusion-display gene is integrated within the viral genome) with a surrogate system, employed herein, that exogenously provides the fusion-display protein for addition to phage capsid. It is easily possible to produce fully coated lambda display particles (LDP) serving as single epitope vaccines (SEV), or antimicrobials, or to produce partially coated LDP without any complex bacteriophage genetic engineering, making the system available to all. The potential to build vaccine vector phage particles (LDNAP) comprising essentially sheathed DNA vaccines encapsulated within an environmentally protective capsid is described. LDNAP are produced by introducing a cassette into the phage genome either by phage–plasmid recombination or cloning. The cassette carries a high-level eukaryotic expression promoter driving transcription of the vaccine candidate gene and is devoid of plasmid resistance elements.

## 1. Introduction

### 1.1. Phages

Phages are bacterial viruses with either double-stranded (ds) or single-stranded DNA or RNA genomes. Phage particles, representing a genome encased within a capsid of protein, represent the predominant form of genetic biomass on Earth. Those with ds DNA genomes are estimated at >10^31^ [1], or by simplistic analogy 20 billion particles per mm^2^ of the earth’s surface. Overall, many phage types represent an enormous and diverse gene pool constantly changin, attributable to recombinational events arising within an infected host cell, for example by exchanging parts (a) with prophage (or prophage fragments) integrated within the chromosome of the infected cell, (b) with extrachromosomal elements (as plasmids or phage genomes) within the host cell, or (c) with coinfecting phage genomes injected in parallel into the host cell. Phage can encode genetic traits (lysogenic traits) that allow the phage genome to stably coexist within its host cell without killing it (i.e., the temperate phage). These temperate phage genomes undergo a decision, upon entering a host cell, to either stably associate within the host or undergo a lytic cycle to kill the host and release progeny as phage particles. Other phage types (the lytic phage) lack lysogenic traits. They may encode highly toxic substances causing death/lysis of the infected cell and only undergo a lytic cycle. Humans have evolved in the constant presence of this biomass and yet there is no known human disease linked to direct phage exposure, even though phages clearly play a role in the evolution of bacterial pathogens [2,3,4,5,6,7,8].

### 1.2. Use of Phages as Therapeutic Agents

Basic approaches include phage therapy (PT) and phage display (PD). The rationale for PT assumes (a) that predator phages (perhaps a cocktail of different infectious types) that infect a form of pathogenic bacterium can be selected and purified from natural sources (e.g., from sewage) and concentrated, and (b) a cocktail of phages (with differing infective mechanisms) can be introduced within a complex milieu (animal infected by pathogenic bacterium) and effect a reduction or elimination of the pathogen. PT was first employed in 1919, shortly after the discovery of bacteriophages [9,10,11,12,13,14,15,16,17,18], and a few recent strategies for PT in wound care and infection, which is beyond the scope of this manuscript, may be explored further in references [19,20,21,22]. PD requires strategies for fusing the coding region for a protein, polypeptide, or peptide with a phage surface capsid gene and then displaying the hybrid protein on a virus capsid. The display of peptides and proteins on the capsids of *filamentous* or *lytic* phages is a powerful technique for the identification or targeting of specific ligands and can be adapted to generate vaccine reagents [23,24,25,26,27]. Gupta et al. [24] have suggested the use of display vehicles for targeted gene delivery, in vivo diagnostics, isolation of tissue-specific peptides, tissue imaging agents (in vitro and in vivo), cell targeting [28], studying the specificities of immune responses of patients with various diseases, cancer therapy, autoimmune disorders and age-related conditions, design of diagnostic assays and therapeutic vaccines, as bio-detectors for a variety of threat agents (viruses, bacteria, spores, and toxins), as drug and vaccine delivery to specific cells, and for applications involving the use of surface display vehicles as biocatalysts.

### 1.3. Phage Display (PD) Systems

In PD the fused polypeptide, perhaps with activity as a ligand, or antigen, can theoretically be designed to join either the NH_2_- or COOH- terminal ends of a capsid protein. Display density refers to the extent to which the fused capsid protein can be added to the capsid. Virus particles with only one or a few gene-fusion display proteins per capsid have low display density, whereas particles displaying perhaps hundreds of fusion proteins have high display density; thus, display density can vary from less than one per collection of particles to greater than hundreds per particle. Display densities as low as one or fewer per particle are important in isolating binder particles with very high affinities for a target ligand; displays of three to ten copies of the fusion-display protein are sufficient to allow isolation of binders to bait when the strength of interaction is in nanomolar to micromolar range. However, for the study of weaker interactions (micromolar to millimolar range), the display needs to be on the order of a few hundred fusion proteins per particle [24]. “Landscape vehicles” [24], i.e., those which have a very dense display of the candidate peptide on the capsid surface of the phage particle, may be better immunogens for use in live vaccine development and as vehicles for biocatalysis and bioadsorption.

The two main types of PD systems are *filamentous* and *lytic* as reviewed in [29]. Filamentous PD was introduced by Smith in 1985 with the typically low density display of peptides fused to one of the low copy capsid proteins at the ends of linear filamentous phage, e.g., M13 [30]. This method of PD, referenced many thousands of times, and with numerous clever variations for either cloning the fusion protein within the vector genome or the use of surrogate systems to produce the fusion protein, is based on M13 and related filamentous phages that are released from infected cells by extrusion of phage particles through the cell wall without producing cell lysis. An M13-encoded fusion capsid protein must traverse the bacterial cytoplasmic membrane and then be assembled on the phage coat/capsid within the periplasm. This can hinder the display of highly hydrophilic proteins. In contrast, *lytic* PD employs either temperate phages, as λ (the focus of this study), or true lytic phages as T4 [27,31,32,33,34] and T7 [35] that release (burst) from infected cells by lysing them in terminal stage of the phage growth cycle. Phage particles released by cell lysis can theoretically display hundreds of capsid fusion proteins per particle and in some systems the capsid fusion display protein, prepared separately, can be added to a procapsid structure in vitro, e.g., both the *hoc* and *soc* genes encoding T4 capsid proteins are dispensable and can be added exogenously [31,32,33,36,37].

The display of peptides and proteins on the capsids of filamentous or lytic phages is a powerful technique for the identification or targeting of specific ligands and can be adapted to generate vaccine reagents [23,24,25,26,27].

### 1.4. λPD

During phage lambda (λ) morphogenesis its genomic ds DNA is packaged into a preformed prohead which expands during packaging, exposing sites for increased binding of the capsid head decoration protein gpD (11.4 kDa) to the underlying molecules of protein gpE making up the prohead [38,39,40]. In the process of prohead expansion into a stable mature λ head, about 420 gpD monomers are enzymatically added to the prohead via conversion into 140 gpD_3_ trimers, which are affixed to 20 icosahedral faces of the mature particle. The gpD_3_ trimer was shown to stabilize the capsid head shell during packaging of the lambda genome [41,42]. The addition of gpD to the prohead is dispensable when the 48.5 Kb λ genome includes a deletion of at least 18% [43]. Cryo-electron microscopy and image reconstruction have shown that the side of the gpD_3_ trimer that binds to the capsid is the side on which both the NH_2_- and COOH- terminal ends reside. Despite this orientation of the gpD trimer, fusion proteins connected by linker peptides to either terminus bind to the capsid, allowing protein and peptide display [44].

While fusions to gpV, the tail tube protein have been reported [45,46], most λ PD reports involve engineered recombination into the λ genome fusing the coding region of a foreign protein/peptide/polypeptide (<) with gene *D*, making *D*< [47,48,49,50,51,52,53,54]. Each of these studies anticipate the formation of a viable phage where the chimeric gpD< protein retains gpD functionality and “<“ has been fused either to the NH_2_- or COOH- terminal ends of gpD [55], or internally within the *D* coding sequence [46]. For gpD< to function similarly to gpD it should not interfere with (i) the interaction of gpD or gpD< monomers to form a trimer or (ii) the incorporation of gpD_3_ or gpD<_3_ trimers on the surface of the λ particle capsid head. If the binding of gpD< is challenged, the expansion of the head is blocked, negating full packaging of the lambda genome, stabilization of lambda particles, and the formation of viable phage particles. Diverse proteins have been fused at the N- or C-terminal ends of gpD, including β-galactosidase protein [55], Cry1Ac toxin from *Bacillus thuringiensis* [56], and the ligand-binding domain of the human peroxisome proliferator-activated receptor gamma [LBDPPARγ] [57]. In the latter example, the solubility of the LBD of PPARγ, which is very prone to protein aggregation, was dramatically increased, indicating that generating a gpD-fusion may improve the solubility of proteins which normally clump and precipitate in *E. coli*. (See [58] on using lambda display as a powerful tool for antigen discovery). It is likely that the extent to which genomically encoded gp*D*< fusion proteins can form gpD<_3_ trimers on lambda capsid heads is limited/varies [59,60]. The numbers of fusion proteins displayed by λ appear somewhat size-dependent: small peptides of about 10 residues were present at as many as 405 copies per particle, with lower display densities seen for larger gpD< fusions; however, overall, phage display on lambda was reported two to three orders of magnitude higher than was displayed by pIII and pVIII fusions on M13 [59].

From the late 1970s the mechanism for packaging of phage λ DNA was beginning to be understood, including recognizing the components of the phage terminase that cleaved λ DNA at its *cos* site needed for initiating phage DNA packaging into the procapsid. This information led to strategies for packaging λ DNA in vitro [61,62,63,64,65], permitting the manipulation and packaging of genetically engineered λ DNA into mature phage particles. Ansuini et al., 2002 [47] engineered a λ phage with two copies of *D* for the purpose of circumventing the presence of having only a single marginally functional gpD< fusion product available for phage display, by provisionally being able to supplement capsid formation dependent on gpD< with wild-type gpD. They developed a clever licensed vector λD-bio containing two copies of capsid gene *D*. One copy of *D* included a suppressible nonsense (amber) mutation, where gpD would only be produced when the phage was grown in *E. coli* cells encoding a nonsense suppressor tRNA, and the gpD produced would only be changed from wild-type gpD by the substitution of the suppressor amino acid at the site of the nonsense mutation. The λD-bio contained a second *D* encoding the gpD< fusion gene. This gpD fusion gene included after its *D* coding sequence the in-frame cloning sites *SpeI* and *NotI* with an included ochre termination codon followed by a 13-amino-acid sequence recognized by the biotin holoenzyme synthetase BirA, and then a second termination signal. Cloning of out-of-frame DNA fragments between *SpeI* and *NotI* nullified synthesis of the downstream peptide recognized by BirA, whereas selection for clones with open reading frames inserted between *SpeI* and *NotI* was made possible by streptavidin affinity chromatography binding BirA. This system and its derivatives [46,66,67] enabled phage display by co-expression of functional gpD and gpD< gene fusions; however, the recombinant phage tended to accumulate mutations able to block gpD< fusion protein expression, resulting in better phage particle production [46] by omitting synthesis of the gpD fusion. Recalcitrant gene fusion combinations of gpD< blocking phage display were identified to be incompatible with the assembly of stable phage particles [68,69], yet the recalcitrant property of gpD< was found suppressible, permitting its display, if a surrogate plasmid system that simultaneously produced gpD was added.

Heat-induced λ capsid disassembly yields only soluble gpD monomers, not gpD trimers, suggesting the trimer rapidly dissociates when not attached to the λ head [70] and gpD remains soluble (does not aggregate) in high molar concentrations [44]. In contrast, in similar treatment, the major capsid protein gpE is easily precipitated and likely exists as aggregates [70]. Data from phage display by co-expression of functional gpD and gpD< gene fusions tend to suggest that gpD_2_gpD< or gpD<_2_gpD, or gpD<_3_ trimers can likely form on the λ head [46]. In vitro complementation has been employed to determine if N-terminal or C-terminal gpD protein fusions will add to a lambda capsid head devoid of gpD. In this system, 10^8^ purified λ *Dam15* phage particles (devoid of gpD), with 79.5% of the wild-type genome size, are mixed with purified gpD or gpD fusion proteins, to permit addition to the capsid, and then subsequently treated with EDTA (results in expansion of the phage heads devoid of gpD), chelating away Mg^2+^. The particle reaction mixture is then mixed with *E. coli* cells able to suppress the *Dam15* mutation and resulting phage plaques are counted. Just about any type of N- or C-terminal gpD fusion has been shown to complement for gpD in this assay [44,46], where “complementation refers to rendering the phage resistant against EDTA treatment by binding the protein in question to the [purified] λ*D*^−^ phage” particles [44]. Though I may be missing something, these data omitted that the purified λ *Dam15* phage particles (devoid of gpD) with 79.5% λ genomes are already resistant to EDTA, and when plated on an *E. coli*, suppressor strain will form viable phage plaques (whether or not they were pretreated with EDTA). The critical argument for complementation was that treatments with gpD deleted for 14 N-terminal amino acids complement very poorly. However, this could also mean that the NΔ14-gpD protein negatively complements in a reaction that does not require any gpD.

## 2. Materials and Methods

### 2.1. Microbiological Techniques

The methods utilized can also be found described in detail in the Section 2 of [71,72,73,74,75,76]. Cultures and phage lysates were prepared in Lysogeny Broth (LB) (10 g Bacto tryptone, 5 g Bacto yeast extract, 5 g NaCl per liter), and made 50 ug/mL with ampicillin. The bacterial and bacteriophage strains employed are described in [71], except for strain C3026H available from New England Biolabs, with reported genotype F’ lac, pro, lacI^q^/Δ(ara-leu)7697 araD139 fhuA2 lacZ::T7 gene1 Δ(phoA)PvuII phoR ahpC* galE (or U) galK λatt::pNEB3-r1-cDsbC (Spec^R^*, lacI^q^*) *ΔtrxB rpsL150*(Str^R^) *Δgor Δ(malF)3*. What is also described is the following: in vivo infection-complementation methodology/assays using transformed cells, considerations relating to complementation versus reversion assays, versus *D-*amber marker rescue, and considerations relative to assay temperature for complementation assays.

### 2.2. Phage Lysate Preparations Methodology for Preparing gpD< Display Phages

#### 2.2.1. Single Phage Infections

Cells (grown overnight to stationary phase) were pelleted and resuspended in TM buffer [0.01 M Tris, 0.01M MgCl_2_, pH 7.8]), mixed and incubated 30 min at 35 °C at MOI of 0.04 with λ*imm434cI#5Dam123* (reversion frequency = 4.2 × 10^−6^). The mixtures were then pipetted into LB-Amp50 medium (held at the intended lysate incubation temperature) made 0.001M in MgCl_2_, and 0.01M in Tris, pH 7.8, and shaken until culture lysis in a heated water bath.

#### 2.2.2. Trivalent Infections

This comprised a mix (0.34 mL of each) of the three expression cell types grown at 30 °C overnight, pelleted, concentrated two-fold, and suspended in TM buffer. Each separate cell aliquot was infected at MOI of 0.04 with λ*imm434cI#5Dam123*, held at 35 °C for 30 min, and the mixture pipetted into a liter of LB broth (done in duplicate, yielding two liters of lysate). The cell titers for the culture cells were determined simultaneously as follows: 594[gpD-RBD#1] (1.14 × 10^9^ cfu/mL), 594[gpD-RBD#2] (5.95 × 10^8^ cfu/mL), and 594[gpD-RBD#3] (fusion of Dcoe at C-terminal to spacer and 11 amino acids (#496–506) from 2019-nCoV capsid receptor binding domain) at 1.38 × 10^9^ cfu/mL.

#### 2.2.3. Single Burst Infections

Single colonies of the host cells are inoculated into four two-liter flasks, each with one-liter LB-Amp50 culture medium. The cultures are incubated with shaking to reach stationary phase and the cells from each flask are pelleted and resuspended in 1/10th volume of fresh LB-Amp50 medium (about 1.5 × 10^10^ colony-forming units (CFU) per ml). The new cultures are incubated with shaking at 30 °C for 30 min and then infected with λ*imm434cI*#5 at a multiplicity of infection of 3 to 5. The phage cell mixture is held at room temperature for 15–30 min without shaking. Then, the infected cells are transferred into four six-liter flasks, each with two liters of LB-Amp50 culture medium prewarmed to 39 °C. Raising the cell temperature from 30° to 39 °C permits expression of the *D-fusion* gene from the plasmids present in the cells. The culture absorbance is monitored at A_575nm_ for 60–90 min, and when it drops to about 0.05 the flasks are moved from shaker to a cold room, the cells are pelleted, and the pellets are discarded. Of note, normal phage lysates are made by a two-step procedure, where initial culture cells outnumber infecting phage by ten-or-more fold. However, in preparing display phage, an objective is to avoid (as much a possible) released display phage from reinfecting growing cells and consequently being lost in the lysate by having the phage capsid affixed to the outside of the growing cells.

### 2.3. Banding Phage in CsCl

The supernatant from a cell lysate (after pelleting cell debris) is pooled into a carboy, and solid polyethylene glycol (PEG, average molecular weight 6000–7000) is added to 4% along with solid NaCl to 0.5M. The additions are shaken into solution and the container is held overnight at about 4 °C. The treated lysate is spun at 8000 revolutions per minute for 15 to 20 min in a 6-liter capacity JA 9.1000 Avanti rotor and the supernatant is discarded. The pellets are each resuspended at 4 °C in about 1 mL phage buffer, adjusted to a refractive index of 1.382 with solid CsCl, and spun at 30,000 in a Ti70 ultracentrifuge rotor (Coulter-Beckman) overnight at 4 °C. The collected bands are pooled and re-banded in a Ti60 rotor. The banded phages used for protein gel analysis or other immunological assays are dialyzed in phage dialysis buffer (0.3 to 0.5M NaCl, 0.01M Tris, 0.05M MgCl_2_, pH 7.8) and then twice against 3000× volume of PBS (0.0036M KCl, 0.0014M KH_2_PO_4_, 0.136 M NaCl, 0.004MNa_2_HPO4, pH 7.4). When only one liter (or less) of a lysate from each infection in needed the PEG step is omitted, lysate is subjected at differential centrifugation, first at 8000 rpm to remove remaining nonlysed cells and large cell debris and then spun in large polycarbonate tubes in a 60Ti rotor (Coulter-Beckman) for 24 hrs at 30,000 rpm to pellet phage (without PEG). The pellets are resuspended by covering them with 0.5 mL of buffer (0.3 NaCl, 0.01M Tris, 0.01M MgCl_2_, pH 7.8), allowing them to soften overnight at 4 °C. The resuspended pellets are adjusted with solid CsCl to a refractive index of 1.382 and the phages are banded in an ultracentrifuge as described above. Any remaining free protein in the phage pellet is excluded from the gradient and forms a thin band above the CsCl solution.

### 2.4. Determining the Toxicity of the gpD-Fusion Constructs by Measuring EOP

The toxicity of gpD< fusion protein expression from pcIpR[GOI]-timm plasmids is readily determined by growing transformed *E. coli* cultures at 30 °C, then diluting and spotting aliquots on LB or LB-Amp50 agar plates, and incubating the inverted plates in incubators with temperatures held at, e.g., at 30, 35, 37, 38, 39, 41, or 42 °C. The EOP represents the average number (of four or five aliquots) of colonies formed per plate per applied aliquot spot, divided by that obtained for parallel assay plates incubated at 30 °C. Some of the gpD< proteins expressed at 42 °C are highly toxic (EOP’s of <0.0001), compared to cell growth at 30 °C (no gpD< expression), base EOP = 1.0.

### 2.5. Plaque and Colony PCR Techniques

A phage lysate or cell aliquot is stripped out on an agar overlay plate with fresh host cells, or a culture aliquot is streaked onto agar plates to obtain isolated plaques or colonies. Either a single plaque is removed using a sterile wide-tipped (Aardvark) micropipette tip or a tiny amount of a fresh colony is picked (a barely visible amount) with a sterile 10 μL micropipette tip. The plaque material, or bit of the single colony is transferred into 50 μL TE* buffer (10 mM Tris + 0.1 mM Na_2_EDTA) in a 0.5 mL microcentrifuge tube. The solution is gently mixed and then incubated at 37 °C for 1 h. The tube with sample is heated in a thermocycler at 96 °C for five minutes to disrupt contents and then centrifuged for one minute at 12K rpm in Eppendorf microcentrifuge to pellet any debris. An amount of 10 μL of the supernatant is mixed with 10X PCR buffer (if it does not contain MgCl_2_, 3 μL of 50 mM, MgCl_2_ wasis added) along with 16 μL of a 1.25 mM solution of the four deoxynucleotide triphosphates, plus 100 picomoles of each of the 2 primers, and sufficient sterile HO to make 100 μL total, plus 0.5 μL of Taq DNA polymerase at 5 units/μL. (For 50 μL reactions decrease amounts by half). The tubes are placed in a thermocycler and run for 25 cycles of denaturation/annealing/primer extension. Many of the phage- and colony-handling genetic techniques are described in [76]. DNA preparations from either plaques, plasmid-transformed colonies, or DNA bands (extracted from acrylamide gels) were submitted to DNA sequencing services and the sequences obtained from four to six representative clones of plaques, colonies, or gel bands were scrutinized for accuracy before the phage, plasmid, or colony was used in an experiment.

### 2.6. Nonradioactive DNA Probe Labeling and Hybridization to Identify Recombinant Phage Plaques

The notched nitrocellulose blots showing plaque recombinants hybridizing to an EGFP probe were placed over the original blotted agar overlay plate. The regions with dark spots were pierced with a large bore needle, then the soft agar overlay region under the pierced spot was sucked out with a large bore hypodermic needle (or/followed with Aardvark large bore plastic micropipette tip). The tiny contents were transferred to 0.3 mL phage buffer (0.01M Tris HCl, pH 7,8, 0.01M NaCl, 0.001M MgCl_2_), in a 1.5 mL micro centrifuge tube, incubated 30 min, pelleted 1 min at 12K rpm in an Eppendorf micro centrifuge, and aliquots of the supernatant were stripped or overlaid on agar overlay plates with fresh *E. coli* host cells. Individual plaques arising were picked again into 0.5 mL phage buffer, and 0.1 mL aliquots were mixed with 2.5 mL top agar with 0.1 mL of fresh *E. coli* host cells poured over fresh LB-Amp50 agar plates. These plates were then blotted with nitrocellulose filters and hybridized with a DNA hybridization probe. Some single plaque isolates (membrane on right side) showed that all the plaques derived from a single plaque isolate included the recombinant EGFP cassette.

In these assays, phage plaques (with released DNA) on agar overlay plates were blotted to nitrocellulose filters and then hybridized with digoxigenin (DIG)-tagged EGFP PCR DNA. We used the DIG System for nonradioactive labeling and detection of nucleic acids. The randon-primed DNA probe labeling produced digoxigen-dUTP tagged EGFP DNA probe. It is bound during hybridization to plaque DNA, bound to the nitrocellulose membrane, and the bound probe is detetected as a purple color precipitate linked to anti-DIG alkaline phosphatase conjugate antibodies. (All DIG reagents for probe labeling and detection of hybridization of labeled probe to membrane were obtained as a commercially available kit from Roche Applied Sciences, #11093657910).

### 2.7. E. coli Strains, Phages, and Plasmids Employed

The plasmids utilized herein are described in Table 1. The sequence and details regarding the backbone prokaryotic expression plasmid pcIpR-timm = pcIpR[GOI]-timm from which all the gpD< fusions were made is described in detail in [73]. Considerations related to making the eukaryotic expression plasmids by this laboratory, borrowing from pMZS3F, a mammalian expression vector, (6309 bp) [77] received from J.F. Greenblatt, University of Toronto (which contains several parts derived from pCI-neo (Promega)), are described in the Supplement: Plasmid Construction Example.

### 2.8. Genetic Techniques

Each of the plasmids constructed were made ordering Gene-blocks of the designed sequences from Integrated DNA Technologies (IDT), Coralville, ID, or were prepared by PCR amplifications employing primer combinations that included requisite terminal restriction site(s). These were also received from IDT. Recombinant plasmid preparations were transformed into *E. coli* [85] and then individual colonies of the unique isolates (usually about five) were subjected to colony PCR and the results for each clonal isolate were sent for DNA sequence analysis. Each of the isolated plasmid constructs from clones employed were verified for DNA sequence integrity. The techniques used for colony PCR, plaque PCR, DNA sequence analysis, and acrylamide gel electrophoresis were previously described [76,77,85]. The mutation *imm434cI*#5 in phage λ*imm434cI#5* (defective in lysogeny) was recombined into *λimm434Dam123* (capable of lysogeny) and single plaque lysates of the recombinant phage *λimm434cI#5Dam123* (A2a, A2b, G1a, G1b), each defective for lysogeny, were employed in future infections (Figure 5 tubes 3–6) to improve lysate titer.

### 2.9. Protein Gels and Western Blots

The SDS denaturing gels and staining methods used for protein analysis steadily improved during course of this study. An early 12% acrylamide SDS denaturing gel, running out an *E. coli* protein extract was stained with Coomassie Blue. The remaining protein gels employ Invitrogen Novex Pre-cast 10–20% Tricine-SDS slab gels (Thermo Scientific) run about one hour at 130 V. The gels were stained with Bio-Rad Oriole fluorescent gel stain and copied in a Bio-Rad Chem Doc using UV light excitation specific for Oriole stain, which proved both easier, more sensitive, and more reliable than an attempted silver stain (SilverQuest Silver Staining Kit) in our hands. The standards employed included Bio-Rad Precision Plus Protein Standards (10 to 250 kDa) and ThermoScientific Low Range Protein Ladder (2, 6, 10, 15, 25, 40 kDa). The gels were electrophoretically blotted overnight at 4 °C to PVDF membrane using a Bio-Rad transfer cell, and then blocked and incubated with primary anti-His-gpD mouse antibody (1:1000) [72], rinsed, incubated with secondary rabbit anti-mouse IgG (1:1000) conjugated with horseradish peroxidase, and developed with Bio-Rad enhanced chemiluminescence (ECL, luminol and H_2_O_2_) exactly as described (Protein Gels and Western blots, [73]). The developed gel blots reacting with His-gpD mouse antibody were recorded using Bio-Rad Chem Doc. Note that the Western blot for expression of gpD-CAP gene fusion from pcIpR-D-CAP-timm plasmid shows the gpD-CAP fusion protein expressed in *E. coli* cells reacting with anti-Porcine Circovirus 2 polyclonal antisera obtained from a gnotobiotic pig, using procedures described in [74,75].

### 2.10. Fluorescence Assays for Expressed gpD-GFPuv

Cultures were inoculated from single CFU of 594 into LB and 594 *E.coli* [pcIpR-D-GFP-timm] into LB made 50 ug/mL with ampicillin and grown overnight (ON) at 30 °C. Larger parallel subcultures (150 mL LB) were made by inoculating 7 mL from each ON culture to LB and then shaken at 30 °C until A_575_ = 0.2, whereupon they were moved into shaking water baths at 37, 39, or 42 °C. Aliquots were withdrawn prior to transfer and at 20 through 180 min post-induction (3 mL to measure absorbance and 5 mL was transferred and held on ice). Following the last sample removal, all the aliquots were centrifuged at 8000 rpm for five min, decanted, and the cell pellets were resuspended in 5 mL of 1X PBS (10X = 80 g NaCl, 2 g KCl, 14.4 g Na_2_HPO_4_.2H_2_O, 2.4 g KH_2_PO_4_ per liter distilled H_2_O, pH 7.4), pelleted, and resuspended in 1 mL PBS (to 5-fold concentration). Three aliquots (100 μL) of each of the resuspended cell pellets were assayed for fluorescence using a Photon Technology International, Model LPS 100, Serial No:0177, MFG 01/11) spectrophotometer. The excitation wavelength was set at 395 nm and emission fluorescence measured at 509 nm. Triplicate assays for each time point were made and the results averaged. Values for plasmid cells represent the average of triplicate assays subtracting the triplicate average for cultures of 594 transferred in parallel to 37, 39, or 42 °C. (We appreciate that the fluorescence of cells grown in minimal medium is lower, but the fluorescent assays for cells grown in LB were undertaken to parallel with the other assay procedures employed herein). To prepare cell lysates, the cell pellets in 1 mL PBS were pelleted at 4500 rpm for 10 min and the supernatant was decanted. The pellet was resuspended in 0.15 mL lysing mix (minus EDTA) made by adding 1.5 mL 1M Tris.HCl, pH7.8, 45 mL 1M sucrose, 44.7 mL of distilled H_2_O, and 0.8 mL of 10 mg/mL solution of lysozyme made in 10 mM Tris.HCl, pH 7.8. The cell suspension was incubated on ice for 30 min and the spheroplasts pelleted at 4500 rpm for 10 min and supernatant decanted. The pellet was suspended in 1 mL of 0.01M Tris.HCl, pH 7.8, and allowed to sit 15 min at ambient temperature (~21 °C). The cell lysate was transferred into another tube and three 100 uL aliquots were measured for GFP activity as for the intact cell preps.

## 3. Results and Discussion

### 3.1. In Vivo Complementation Assay for gp< Activity

**Figure 1:**
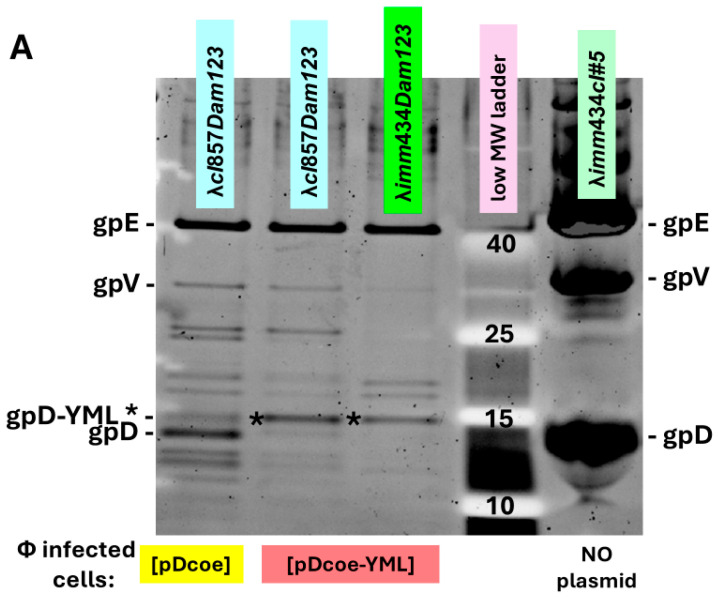

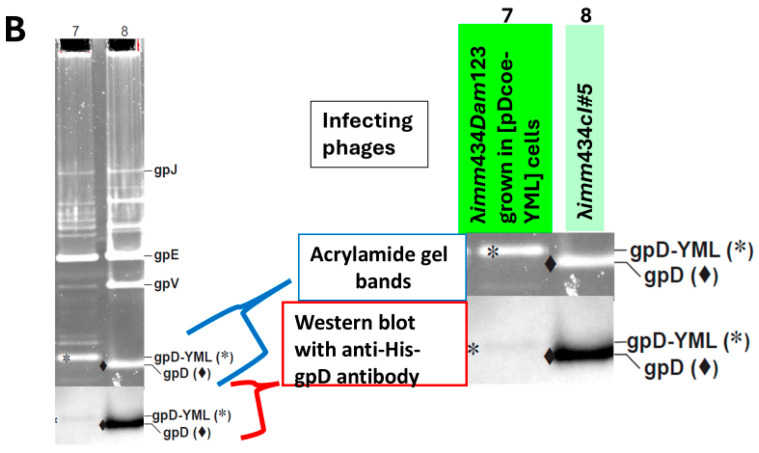
Comparison of gpD and gpD< [gpD-YML] from purified phage particles. Phage particles from separate infections were concentrated by differential centrifugation (first to remove cell debris, second after PEG addition, then collecting and resuspending the pellet). Following CsCl banding (Methods 2.3) and dialysis of banded phage particles, the preps were submitted to denaturing acrylamide gel electrophoresis. The left gel lane in (**A**), and lane 7 in (**B**) show phage particles coated with gpD. The gpD band is lighter than the gpD-YML* band because gpD-YML has 17 more amino acids than gpD (i.e., a the spacer-GGSGAP joined to 11 amino acids GYMLGSAMSRP representing the disease-specific epitope, termed YML, from the sequence of cervid prion pRp protein [73]). (**A**,**B**) represent 10–20% SDS acrylamide gels stained and subjected to Western analysis as described in Methods (Methods 2.9). The lower Western blot (**B**) employed anti-His-gpD mouse antibody with chemiluminescence detection of secondary rabbit anti-mouse IgG (HRP conjugated).

In testing whether gpD< fusions will add to head formation in the presence of gpD, or can complement a deficiency in gpD, it is essential to undertake in vivo complementation assays where the possibility for negative complementation by gpD< is examined. These assays are easily accomplished by (a) assessing the efficiency of plating by gpD^+^ λ phage on cells where gpD< is being expressed in parallel from a surrogate plasmid, and (b) measuring complementation by surrogate expression of gpD< in non-suppressing (nonpermissive) cells infected with λ*Dam* phage, and in parallel, measuring the reversion frequency of λ*Dam* to λ*D*^+^ as a control, found to be less than 10^−5^. Phage particles fully coated with gpD< (where gpD< = gpD-YML in Figure 1A (lanes for λ*cI857Dam123* and λ*imm434Dam123* infecting 594[pcIpR-Dcoe-YML-timm] cells) and Figure 1B (lane 7) are recovered whenever the transformed cells expressing a functional gpD< from a surrogate plasmid are infected with *D-*defective phage. Lanes 7 and 8 are the same as shown in (Figure 6 of reference [72]). In the examples (Figure 1A,B) the host cells are infected at 39 °C where the CI[Ts]857 repressor encoded by the pcIpR-Dcoe-YML-timm plasmid is thermally inactivated, permitting both phages to grow vegetatively. Of note, the *imm434* infectin phage is not sensitive to repression by the λ *imm*λ CI[Ts]857 repressor encoded on the pcIpR-Dcoe-YMY-timm plasmid. In an in vivo assay the influence of gpD< expression on culture viability, i.e., its *intra-*cellular toxicity is easily examined, as is the relative display density of gpD< on phage particles generated by complementation. The Western blot in Figure 1B and in a later figure shows that our His-gpD antibody preps bind well with gpD but bind very poorly with the gpD-YML* band.

**Figure 2:**
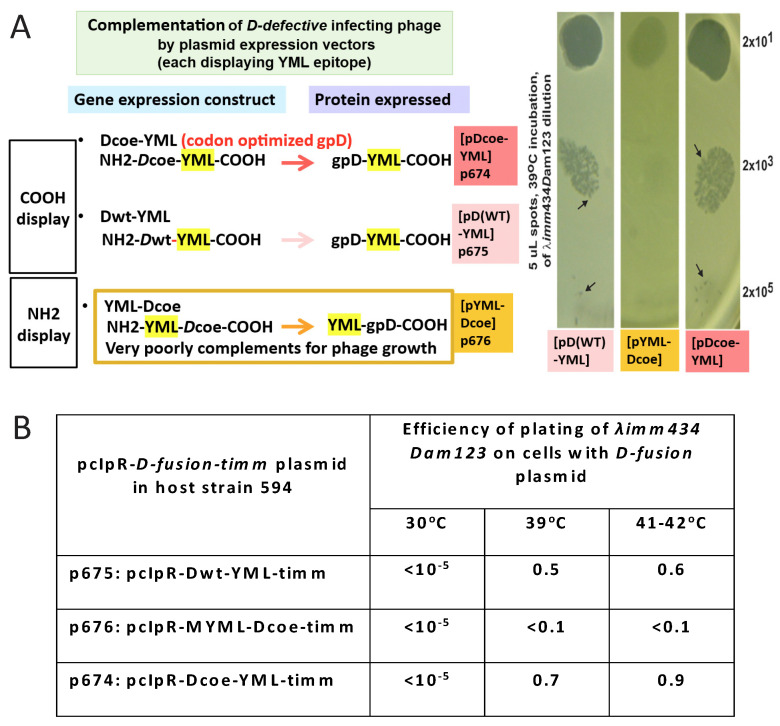
In vivo complementation assay for either N- or C-terminal gpD< fusions ability to substitute for gpD defect on infecting phage. Complementation was evaluated for plasmids that express three different *D*-fusions. The results for 594[pcIpR-Dcoe-timm] and 594[pcIpR-D wild type-timm] are not shown but are identical to the results shown for 594[pcIpR-Dcoe-YML-timm] = strain 594[p674]. Each of the *D*-fusions include an 11-amino-acid (GYMLGSAMSRP, termed YML) single epitope from the sequence of the cervid PrP protein [73]. Plaque formation represents positive complementation, indicating that the expressed gpD< in the infected cells can substitute for the null mutation in gene *D* on the infecting phage’s genome. (**A**) The nonpermissive *E. coli* strain 594 was transformed with plasmids **p674**: pcIpR-Dcoe-YML-timm expressing gpD-spacer[GGSGAP]-YML; **p675**: pcIpR-Dwt-YML-timm expressing gpD-spacer [GGSGAP]-YML epitope; and **p676**: pcIpR-MYML-Dcoe-timm, expressing M-YML-spacer [GSGQ]-gpD. The plating results on 594[**p613**]=pcIpR-Dcoe-timm expressing only gpD are not shown but they were identical to those shown for 594[p674] and 594[p675]. Part A is revision of Figure 5 in reference [72]. (**B**) Efficiency of plating λ *imm434 Dam123* on cells blocked (30 °C) or derepressed for gpD-YML fusion protein constructs. In parallel, the assessed complementation by 594[p676] of λ *Dam123* was less than 10^−5^ at 30, 39, and 41 °C. (Note that λ *imm434 Dam123* is insensitive to the *imm* λ-CI[TS]857 repressor encoded by the plasmids and would be complemented if the p674 or p675 plasmids could express the gpD< fusion at 30 °C.) The efficiency of plating of phage λ *imm434 Dam123* was determined on various host strains with gpD< expressing plasmids by diluting the phage stock 0.5, 10^−1^, 10^−2^, and 10^−3^ and spotting 5 μL aliquots (i.e.*,* 1/200th of phage lysate dilutions) in quadruplicate on agar overlay plates [2.5 mL top agar plus 0.1 mL of stationary phase overnight plasmid transformed cell culture (at about 2 × 10^9^ cells/mL) poured over solidified 30 mL LB-Amp50 agar plates]. After the spots dried (about 15 min) on the overlay plates, they were inverted and incubated in separate incubators held at the assay temperature(s) for 24 h. The plaque forming units per spot per dilution per incubated agar plate were counted and averaged. The efficiency of plating represented the highest average titer per quadruplicate dilution spots giving well separate plaques divided by the original titer of the λ *imm434 Dam123* phage lysate determined on an amber-suppressing *E. coli* host, i.e., on TC600 *supE* cells. The arrows indicate equivalent names for plasmid-encoded gpD-fusion constructs transformed into *E. coli* cells. The complementation assays are color coded: all assays represent one *lambda Dam* phage spotted on agar plates overlaid with cells transformed, and expressing, the different -color-coded- plasmids.

The in vivo complementation assay sensitively detects when gpD< proteins provided by the surrogate plasmid are recalcitrant in suppressing the *Dam* defect coded by the infecting phage (Figure 2). Nonpermissive cells with a surrogate plasmid expressing gpD< with a 16-aminoacid N-terminal addition (NH_2_-YML-Dcoe-COOH = MGYMLGSAMSRP-GSGQ_spacer_-gpD) failed to complement (absence of phage plaques) the infecting *Dam* phage with near wild-type genome size. In contrast, similar expression of either gpD (wild type) or gpDcoe (codon optimized) where the YML epitope is fused to the C-terminal end of gpD (=gpD-GGSGAP_spacer_-GYMLGSAMSRP both complemented well, permitting plaque formation by the *Dam* test phage.

**Figure 3:**
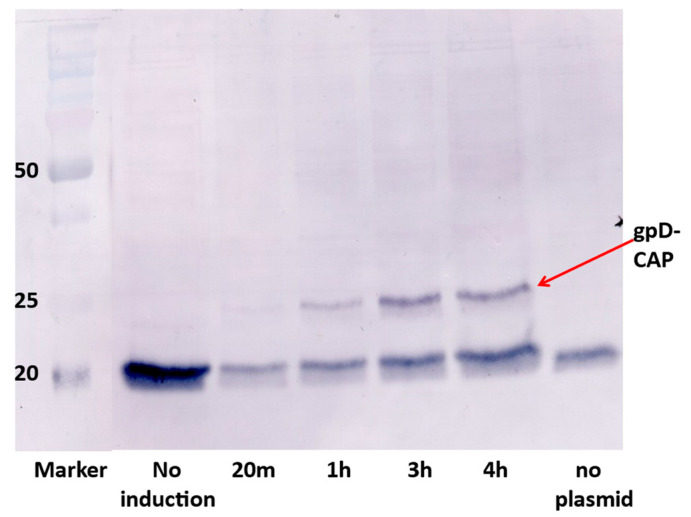
Expression of gpD-CAP gene fusion from pcIpR-D-CAP-timm plasmid in *E.coli* W3350 cells upon shifting cells growing at 30 °C to 42 °C. The gpD-CAP fusion protein represents gpD fused to short spacer (6 amino acids) and joined to 92 amino acids including 4 epitopes from the Porcine Circovirus 2 capsid protein (CAP). *E. coli* cell extracts from each induction time were separated by 12% SDS PAGE. The gel blot was reacted with anti-Porcine Circovirus 2 (PCV2) polyclonal obtained from gnotobiotic pigs (see Methods), as described in detail in [74,75]. The lower band(s) of about 20 kD represents an unidentified protein made by *E. coli* that cross-reacts with polyclonal antibody to PCV2.

The synthetic plasmid pcIpR-GOI-timm expression vector [74] positions the cloned gene of interest (GOI) in the position of λ gene *cro* (on the phage) and is placed just upstream from a powerful λ terminator *tImm* and downstream from λ promoter *pR* (regulated by the plasmid-encoded temperature sensitive repressor CI[Ts]857). Numerous variations of this expression plasmid have been employed [73,74,75,76,85,86] to vary thermally the expression of the cloned GOI from fully off (culture incubation temperatures 25–30 °C) to fully expressed (at 42 °C). When cells with this plasmid are grown at 25 or 30 °C the plasmid-encoded CI repressor can prevent the expression of extremely toxic proteins, peptides, or gpD fusions [72,85]. The utility of expressing gene fusions with gpD is that the fusion protein does not aggregate within the cells or on purification. An example is illustrated in Figure 3 with the 207 amino acid fusion between gpD and epitopes from the Porcine Circovirus 2 capsid protein. We have seen similar results (no aggregation at high concentration) for the 354 amino acid gpD-GFP and similar large fusion proteins.

Thermal regulation of the GOI encoded by plasmid pcIpR-GOI-timm is varied by shifting the transformed cells growing at 30 °C to higher temperatures, as shown in Table 2.

### 3.2. Dual Display

Once it was realized that both gpD and gpD< can be combined on a lambda particle and that in the absence of gpD some gpD< fusions are incompatible with the assembly of stable phage particles [69] it was proposed that “dual” gpD expression systems [74] that eliminated the complex cloning of recombinant *D*-fusions into the lambda vector genome, or into a lambda vector with both *D* and *Dam* genes, could be advantaged. The *D-*fusion could be induced and expressed from a surrogate plasmid in *E. coli* cells while simultaneously infecting the cells either with λ*D*^+^ or λ*Dam* phage. In the first instance, both gpD (generated by infecting phage) and gpD< (expression induced from plasmid) would compete in decorating the capsid head of the infecting phage. In the second situation, the head of the infecting λ*Dam* phage is fully complemented by the plasmid-expressed gpD< fusion protein (Figure 1). In these assays negative complementation by a gpD< (as seen for the N-terminal addition of the YML epitope to gpD, plasmid p676, and pYML-Dcoe) is observed as a reduction in plating of λ*D*^+^ on cells expressing the gpD< fusion protein. Positive complementation is measurable by observing the formation of plaque forming λ*Dam* phage on non-amber suppressing cells expressing a plasmid-encoded gpD< fusion.

**Figure 4:**
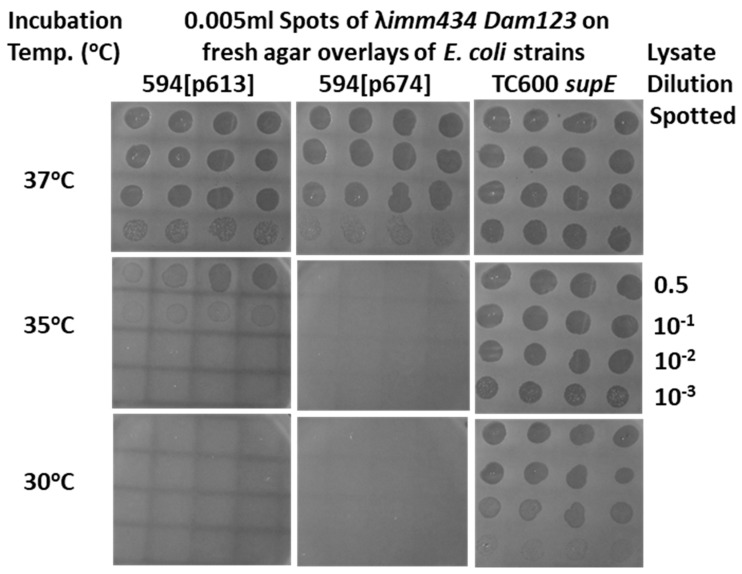
Phage λ *imm434 Dam123* lysate spots (quadruplicate) on nonpermissive (594) and permissive (TC600 *supE*) *E. coli* agar overlay plates as shown in Figure 2A. The objective is to determine the culture incubation temperature where expression of the partially repressed pcIpR-GOI-timm plasmid is able to provide sufficient gpD (p613) or gpD< (p674) to permit complementation of the spotted phage. The lysate dilutions spotted were identical to those shown at 35 °C.

The ability to vary the expression of gpD< is an important aspect of the dual display system, i.e., effectively being able to tune the expression of gpD< from the expression plasmid, pcIpR[GOI]-timm. Even quite low levels of gpD expression from pcIpR[GOI]-timm plasmids at 37 °C (compare to Table 2) can suppress the *D-*defect of an infecting phage, as shown in Figure 4. Previous studies where GOI was the very toxic lambda protein gpP [85] or lambda gpCII [86] showed that their level of expression, as measured by cellular toxicity or complementation, could be varied between culture incubation temperatures of 35 to 42 °C, and the levels of gpD-GFP expression [86], with time, after thermal induction (shifting cultures from growth at 30 °C to 37 °C, 39 °C, or 42 °C) showed marked differences. One might imagine that expressing *more* gpD (or the gpD< fusion protein) is better for complementation, but our experience suggests this would be incorrect. This conclusion may relate to our use of gpDcoe (55 poor codons of *D* optimized for *E. coli*) since lambda evolution likely conspired to limit gpD expression.

### 3.3. Potential Use of Lambda Display Particles (LDP) as Vaccine Reagents

**Figure 5:**
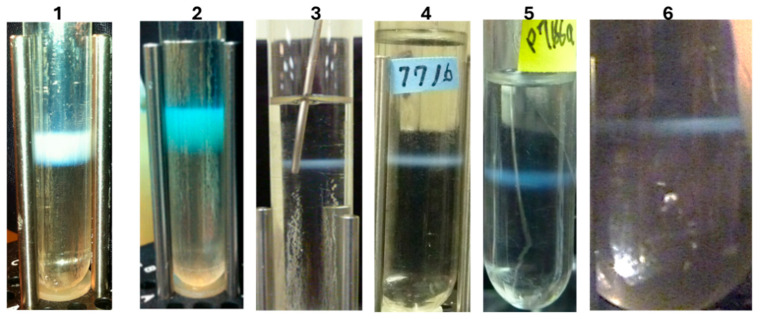
CsCl banding of LDP lysate preparations made by infecting nonpermissive host cells transformed with pcIpR[ ]timm plasmids expressing gpD< gene fusions and carrying out the infections at 37 °C in a shaking water bath. The phage concentration was determined spectrophotometrically after banding, since the phage titer from lysates drops significantly after CsCl banding and storage (see [74]). The calculation was as follows: Absorbance at 260 nm × 20,000 coefficient (=10 mm/2 mm pathlength × 100 dilution × 40 μg phage/OD260 unit) × 9.4 × 10^9^ particles per ug per ml. (The plasmids used to express and prepare the gpD< display phages are described in Methods (Table 1)). **Tube 1.** Display of gpD-BVDV2-E2-Ver2 (251 amino acid fusion protein); band titer 1.265 × 10^10^ particles/μL. **Tube 2.** Display of gpD-BVDV2-E2-Ver3 (241 amino acids fusion protein); band titer 1.56 × 10^10^ particles/μL. **Tube 3.** gpDcoe-YML (C-terminal fusion of gpD to 17 amino acids including YML epitope from prion protein pRp); shown second banding of phage recovered per two-liter lysate, band titer 2.63 × 10^10^ particles per μL. **Tube 4.** gpD-RBD#2 (fusion of Dcoe at C-terminal to spacer and 13 amino acids (#’s 482–494) from 2019-nCoV [87] capsid receptor binding domain for spike protein to human lung epithelial ACE2 protein, Methods Table 1); shown second banding of phage recovered per two-liter lysate: 8.93 × 10^12^ viable particles per 7 × 10^8^ input phage = 1.27 × 10^4^ amplification/input phage to infection. **Tube 5.** gpD-RBD#3 (Methods, Table 1), shown second banding of phage recovered per two-liter lysate: 1.08 × 10^13^ viable particles per 7 × 10^8^ input phage = 1.53 × 10^4^ amplification/input phage to infection. **Tube 6.** Trivalent infection: shown is first banding (larger centrifuge tube than used in lysates used to prepare **Tubes 1**–**5**) of phage output recovered per two liters of lysate: 6.47 × 10^12^ viable particles per 7 × 10^8^ input phage = 9.22 × 10^3^ amplification/input phage to infection. The phage bands seen in Tubes 1 and 2 were produced by a *Single Burst* procedure; the bands shown in Tubes 3 to 5 were produced by *Single Phage Infection* technique; and that in Tube 6 was by the *Trivalent Infection* method, each described in Methods.

**Figure 6:**
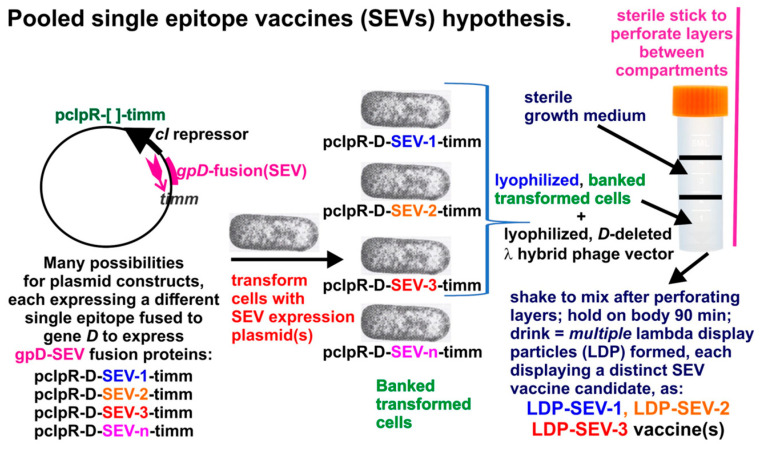
Multiple fully gpD<-coated phage particles (LDP) can be produced in a single infection. This hypothesis is based on the result for simultaneously producing three phage particles, each displaying a distinct disease-specific epitope [87] of the capsid spike protein of the SARS coronavirus (shown in Figure 5, band in **Tube 6**). Of note, the patented, ingestible *E. coli* Nissle 1917 strain (EcN) [GmbH/Pharma-Zentrale GmbH, 58313 Herdecke/Germany] has been described [88] and if a λ-sensitive variant was available/obtainable, it might become a useful host strain.

LDP can be prepared by infecting cells expressing gpD< gene fusion proteins with either λ*D*^+^ or λ*D*^−^ phage lysates (Figure 5). When infecting only with λ*D*^−^ phage, only phages coated with gpD< are produced (see Figure 1). The ability to simultaneously produce multiple LDP in the same infection, as shown in Figure 5 (band in 6), allows the possibility for producing multivalent reagent preparations in the same reaction and permits the hypothesis for rapid production of multiple single epitope vaccine methodology (Figure 6).

**Figure 7:**
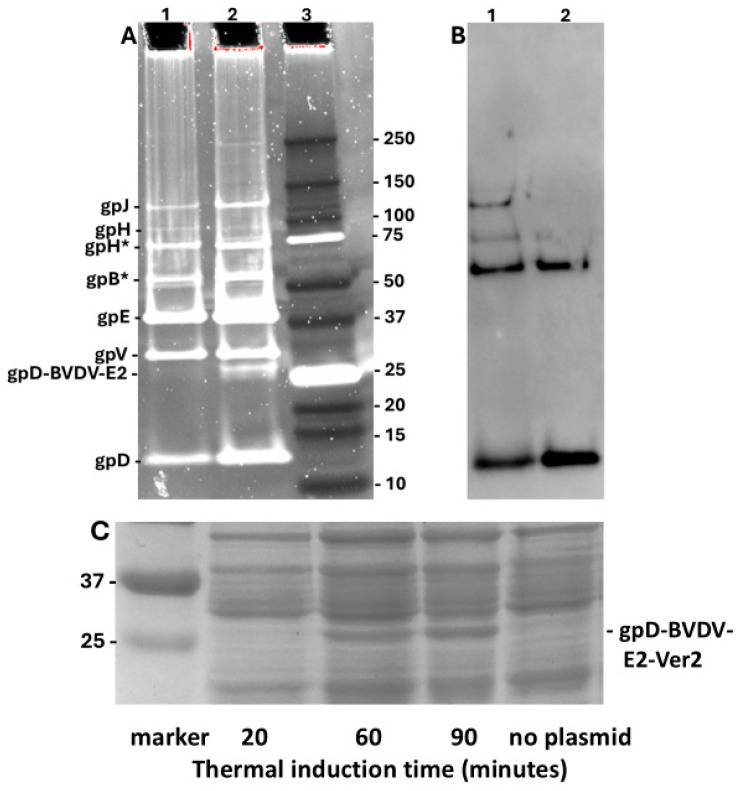
Comparison of phage particles coated with gpD and gpD< (epitopes from Bovine Viral Diarrhea Virus -BVDV2 E2 protein [84,91,92,93,94,95,96] with phage particles obtained without exposure to gpD<. (**A**,**B**). Pre-cast 10–20% Tricine-SDS slab gels (Methods 2.9). **Lane 1.** Phage particles from band shown in Figure 5 (Tube 1): λ*imm434cI#5* infection of nonpermissive W3350 cells with plasmid expressing gpD-BVDV2-E2-Ver2 (see Methods). **Lane 2.** Phage particles from band shown in Figure 5 (Tube 2): λ*imm434cI#5* infection of cells with plasmid expressing gpD-BVDV2-E2-Ver3. **Lanes 1, 2**: Trace analysis (TotalLab software version 2.4 evaluation of UV Trans Illumination and ChemiDoc MP images of the Oriole stain) showed the density/volume of the gpD-BVDV2-E2-Ver3 band (in lane 2) was at least 6.4-fold less than the gpD band in same lane, while the density of similar (very faint) band in lane 2 for the Ver2 display phage was >40 less. **Lane 3.** BioRad marker set 10 kD-250kD. (**B**) Western blot of the gel shown in (**A**), corresponding to lanes 1 and 2, employing anti-His-gpD mouse antibody (see Methods). (**C**) Cell extracts from W3350 cells transformed with plasmid pcIpR-Dcoe-BVDV-E2-Ver2 were grown to mid log in shaking water bath at 30 °C, then shifted to 42 °C to thermally induce the expression of the gpD-BVDV-E2-Ver2 gene fusion protein from pcIpR[GOI]-timm plasmid. *E.* coli cell extracts from each induction time were separated on 12% SDS PAGE, identical to gel employed in Figure 3. The culture for “no plasmid” was collected after heat shift from 30 °C to 42 °C with culture shaking for 90 min. Note: the assignment of faint band for gpH and for gpH* and gpB* is a best guess.

In addition to our earlier studies [75] with the Porcine Circovirus capsid fusion gpD-CAP, we explored whether other complex gpD< fusion proteins could be added to the λ capsid head in the presence of gpD^+^. *E. coli* cells expressing gpD fusions to epitopes from Bovine Viral Diarrhea Virus 2 (BVDV2) E2 capsid protein were infected with λ*imm434cI#5,* i.e., *a D*^+^ phage. The phage particles from CsCl bands shown in lanes 1 (gpD-BVDV2-E2-Ver2) and 2 (gpD-BVDV2-E2-Ver3), Figure 5, were dialysed, diluted, and run on denaturing acrylamide gels, respectively (Figure 7A, lanes 1 and 2). The phage bands are labeled as suggested in Figure 1 of reference [89]. Phage λ*imm434cI#5* used in these infections does not appear to encode either of the tail fiber genes *tfa* or *stf* [90]. Both the Ver2 and Ver3 display phage particles show an extra band (examine region below the band for gpV), presumed to be 248 amino acid gpD-BVDV2-E2-Ver2 (very faint band below gpV in lane 1) and 238 amino acids gpD-BVDV2-E2-Ver3 (easily identified band below gpV, lane 2). Assuming all 420 gpD binding sites are occupied on the phage particles (Figure 5, tubes 1 and 2 obtained from the infections of cells with expression plasmids), possibly 65 binding sites for gpD may be occupied by gpD-BVDV2-Ver3. The Ver3 gpD fusion display protein is 10 amino acids shorter than the Ver2 variant that included an additional (problematic?) 79–88 amino acid loop present in the BVDV2 E2 protein (legend, Figure 7). This suggests increased “recalcitrance” in capsid head binding by fusion protein gpD-BVDV-E2-Ver2 compared with gpD-BVDV-E2-Ver3, since the gel (Figure 7C) clearly shows there is a high level of gpD-BVDV-E2-Ver2 expression from the induced plasmid, implying it was available for binding to the head. The Western blot (Figure 7B) of lanes 1 and 2 (Figure 7A), employing anti-His-gpD mouse antibody (made to the His-tagged-gpD), revealed strong binding to gpD, and to higher molecular weight aggregates of gpD (never previously observed), but there was no binding by the antibody to the gpD-BVDV2-E2-Ver2 or -Ver3 fusion proteins (i.e., to the bands in Figure 7A, lanes 1 and 2) shown slightly above the 25 kD marker (Figure 7A, lane 3).

### 3.4. Evaluating Biological Activity (Cellular Toxicity) of Antimicrobial Polypeptides Fused to gpD

In a previous study [72] we examined whether antimicrobial polypeptides fused to gpD could exhibit biological activity. For this purpose, we fused antimicrobial peptides, including cathelicidins from human (LL37), pig (PR39), or human α- or β-defensins to gpD, and examined whether they retained antimicrobial activity conformation when fused to gpD. We found that fusion to the NH_2_- terminal end of gpD nullified antimicrobial activity for all of them, whereas the same fusions to the COOH-end of gpD each retained strong antimicrobial activity (*E. coli* toxicity). The high toxicity of the fused α- defensin (HD5) and β-defensins (HβD3 and DEFβ126 [deleted for C-terminal 32 amino acid residues]) expressed in 594 host cells suggest that either (a) these COOH-fused defensins undergo the correct formation of three possible intramolecular disulfide bonds after translation within the cytoplasm of wild type *E. coli* cells that were not impaired for synthesis of either thiroredoxin or glutathione (i.e., remained competent in *trxB* and *gor* genes), or (b) that reduced versions of the gpD-linked defensins without disulfide bonds represent the highly toxigenic form of the antimicrobial peptides. There is evidence for the β-defensin HBD1 that only after the reduction of its disulfide bridges does it become a potent antimicrobial [97]. The relative toxicity of gpD-fused α- and β-defensins and cathelicidins expressed in wild-type (594) and in *DsbC trxB* (C3026H) cells where disulfide bridges can form within the cytoplasm was compared (Table 3. In cells (C3026H) where the gpD-fused HβD3 β-defensin and fused HD5 α-defensin could form disulfide bridges, defensin toxicity was eliminated. This did not hold for the DEFβ126 β-defensin that had been deleted for 32 residues at COOH end. Presumably, without these additional 32 amino acids, correct folding to enable disulfide bond formation between any of its 7 cysteins cannot occur. Strain C3026H had no influence on toxicity when either the pig or human cathlicidins were directly fused to the COOH end of gpD (both fusion combinations remained highly toxic), but it could suppress the toxicity of the complex cathelicidin LL37 fusion expressed from plasmid p619 (for which we have no explanation since the additions to gpD do not not contain cysteines). These data support the reported result for defensin HBD1, and suggest that the reduced versions of α- and β-defensins without disulfide bridges (but not the oxidized versions) are required for manifesting their antimicrobial activity.

### 3.5. DNA Vaccines Delivered in Phage Lambda Particles (LDNAP)

The advantages and disadvantages of DNA vaccines, regulatory hurdles and their use against viral diseases, bacterial pathogens, cancer, and immunological responses are reviewed in [98,99,100,101]. The DNA vaccine is usually a plasmid DNA that encodes a disease-specific antigen. The antigen gene is expressed from a broadly active eukaryotic promoter, often derived from the Herpes Cytomegalovirus (CMV). The antigen gene is cloned between the eukaryotic promoter and a eukaryotic termination/polyadenylation (PA) signal. A typical method for delivering a DNA vaccine involves using a gene gun [101,102,103] containing gold particles with the DNA vaccine coated on them. Particles from the gun enter a target cell, where injected DNA enters the cell nucleus [104] and is then transcribed. The mRNA is transported out of nucleus for translation and processing of the expressed protein antigen(s) encoded by the vaccine. The objective is to target antigen presenting cells, especially dendritic cells, that can present the antigenic peptides onto host cell MHC, evoking both cell-mediated and humoral immune responses.

One key disadvantage to DNA vaccines, e.g., compared to protein vaccines, is that the vaccine DNA is degraded by ubiquitous DNase when moved as a DNA reagent into a biological milieu. Phage particles encapsulating a DNA vaccine expression system provide an important improvement over naked DNA, i.e., the DNA packed into the capsid particle (called a sheathed DNA vaccine) is protected from DNase, and particles about the size of phages like λ are readily phagocytized by dendritic cells, increasing the probability that an expressed antigenic gene product is processed by an animal’s innate immune system [105,106,107].

March and coworkers were early pioneers in evaluating mammalian expression cassettes integrated within the lambda genome for the purpose of developing DNA vaccines [108,109,110,111,112]. They were able to use whole λ particles as a DNA vaccine against Hepatitis B showing an immune response in vaccinated rabbits. In this work they linearized plasmid preCMV-HBsAg [Aldverson] and cloned it into a λgt11 DNA vector, generating phage particles for vaccination after λ in vitro packaging of the cloned expression plasmid [110]; similarly, they cloned other eukaryotic expression cassettes, as pcDNA3 or Stratagene plasmid pBK-CMV into the Stratagene cloning vector λZAP or again into λgt11.

These early attempts potentially suffered in two ways. First, all of the plasmid eukaryotic vectors incorporated into phage particles included antibiotic resistance genes that were moved via DNA vaccine construction into animal hosts. Gene transfer of antibiotic resistance into a eukaryotic host during vaccine administration seems undesirable. For example, Stratagene plasmid pBK-CMV encodes kanamycin/neomycin resistance and pcDNA3.1/myc-His(-)A (or B or C variants) encode both ampicillin resistance gene for selection in *E. coli* and neomycin resistance gene for selection in eukaryotic cells. Secondly, eukaryotic gene expression from the plasmids they employed was likely quite low (by a factor of greater than 20) compared to expression from the pCI-neo (Promega) cloning vector [Promega Notes Magazine 51:10 (1995)].

With these limitations in mind, we chose to design plasmids with a powerful eukaryotic expression cassette, where only the expression cassette, without other plasmid components, was moved into a phage λ genome. In short, these phage particles with cloned eukaryotic expression cassettes were designated LDNAPs. It was assumed the LDNAP phage could additionally be grown in cells expressing some gpD< fusion protein, enabling them to target ligands binding to “<“ displayed from the phage. Such particles were termed L2DP (DNA display particles).

Most of the poor eukaryotic expression plasmids included large multiple cloning sites (MCS, about 113 base pairs) and straddling bacteriophage T7 and T3 promoters (184 base pairs), with possibilities for hairpin structures that may limit gene expression. In a set of plasmids, loosely termed pSJH, focusing on pSJH-D’ herein (see Supplement Plasmid Construction Example (for Plasmid p593)), the MCS was reduced to 30 base pairs (*AseI-XhoI-NotI-ClaI*) and the T7 and T3 promoters were eliminated. The eukaryotic expression cassette was designed to be recombined or directly cloned (without any of the plasmid parts or selection genes), into a λ phage hybrid capable of strong plaque-forming ability, i.e., one retaining all the vector’s recombinational potential. (Note: many lambda cloning vectors are highly deleted for nonessential genes and plaque poorly). A comparison of the expression of eukaryotic genes EGFP and Luc2P from the pSJH plasmids to their expression from commercial plasmids is shown (Table 4).

**Figure 8:**
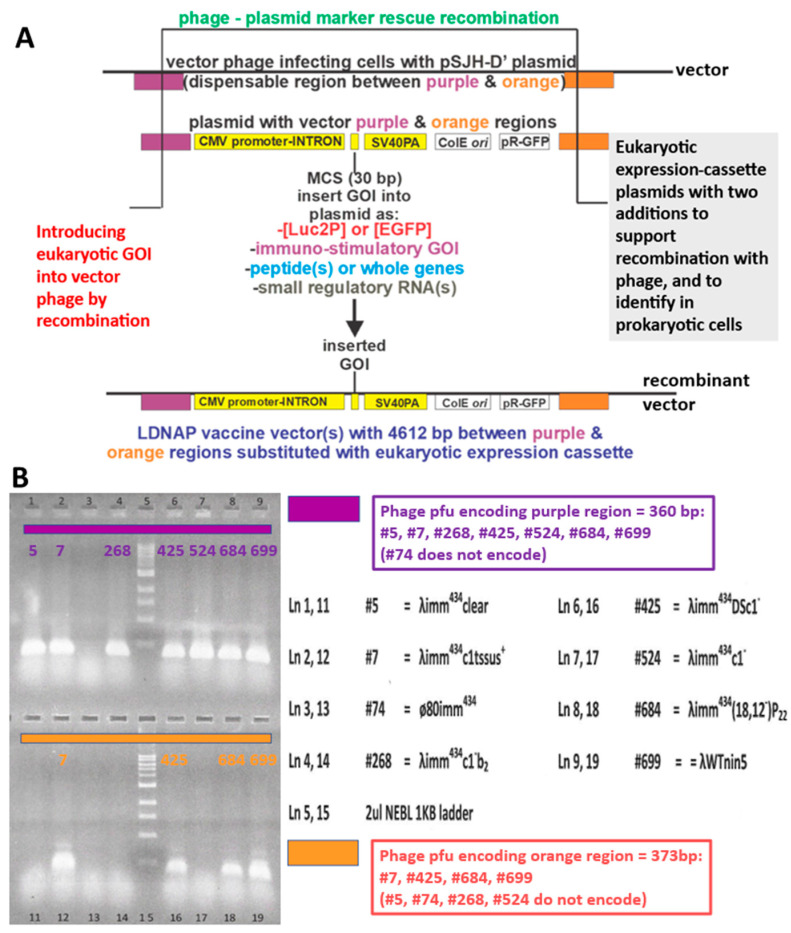
(**A**). Marker rescue replacement recombinational uptake into the infecting phage of the segment between purple and orange regions straddling a eukaryotic expression cassette on plasmid. The infection was carried out in plasmid-transformed *E. coli recD recF* cells, shown to increase phage-prophage recombinational exchange by six-fold [84] above that in wild-type cells. The purple and orange regions were, respectively, amplified by primer sets L-PciI:18968+22 (5′-ata tac atg tcg taa tgt gtg tat tgc cgt tgc-3′) and R-BsiWI:19328-27 (5′-ata tcg tac gca ctg acc tgc tta ctg att tg-3′) and L-AvrII:23937+20 (5′-ata tcc tag gtt gca tgc tag atc gtg a-3′) and R-EcoRIXbaI:24310-20 (5′-ata ttc tag aac agt cat aga tgg tcg gtg g-3′), amplified from λ DNA and cloned into the plasmids in two orientations (same and opposite). (**B**) Plaque PCR assay (Methods) with primers for purple and orange regions to determine if these regions, 360 and 377 bp, respectively, were present in collection of λ*imm*434 phages (each lysate # for individual phage strains is shown) seepotentially used in phage display. Shown in lanes 5 and 15 is a 1 Kb ladder (NEB N0468s):, bands lowest to highest: 0.5, 1, 1.5, 2, 3, 4, 5, 6, 8, 10 Kb) with electrophoresis of bands performed on acrylamide gel.

**Figure 9:**
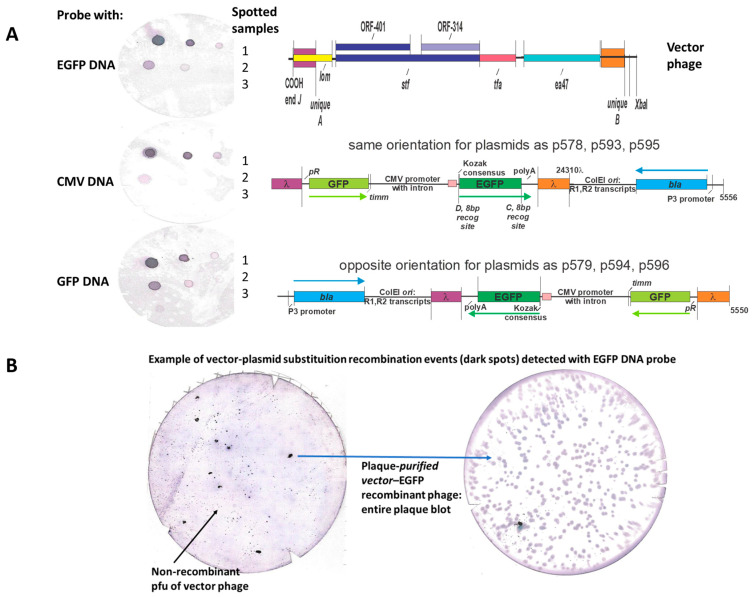
(**A**) Possibilities for in vivo replacement recombination event between purple and orange regions between eukaryotic expression plasmids and the purple and orange sequences on vector phage (Figure 8B #7, lanes 2 and 12). (See Methods, Table 1, for the component organization of p578, p579, and p593-p596 eukaryotic expression plasmids). The phage–plasmid recombination event will substitute the EGFP cassette for phage vector genes as follows: *lom* [113] (mid-through COOH end, just to right of tail gene *J*)- orf 401-orf 314 (comprising tail fiber gene *stf*)-tail fiber gene *tfa*-and gene ea47, all being a region that is fully replaceable in lambda. The membrane blots (left of drawing) show three hybridization assays using DIG-labeled EGFP DNA, CMV DNA, or GFPuv DNA probes with spotted samples on filter membrane as follows: rows 1–3: row 1 (left to right 20, 2, and 0.2 ng plasmid p595 DNA); row 2 (20, 2, and 0.2 ng plasmid p596 DNA); row 3 Phage λ*imm434cI#5* lysate spots, 10^7^, 10^6^, and 10^5^ pfu. (**B**) *Left-side membrane blot:* shows phage plaque recombinants (dark spots identified by DIG-labeled EFGP DNA hybridization probe) from an infection of *E.coli cells* transformed with plasmid p595 (Table 1) with phage vector #7 lysate (Figure 8B). Mostly shown are nonrecombinants (faint plaque outlines) that have not recombined to pick up the EGFP cassette straddled by purple and orange DNA. *Right-side membrane blot:* The arrow drawn from a recombinant spot points to a membrane blot of overlay agar plate where a single plaque isolate purified from some recombinant plaque region (there is no direct correlation implied by the arrow) shows that all the plaques obtained from a single plaque recombinant (left-side membrane) encoded the EGFP gene. The procedures employed are described in Methods for “nonradioactive DNA probe labeling and hybridization”.

**Figure 10:**
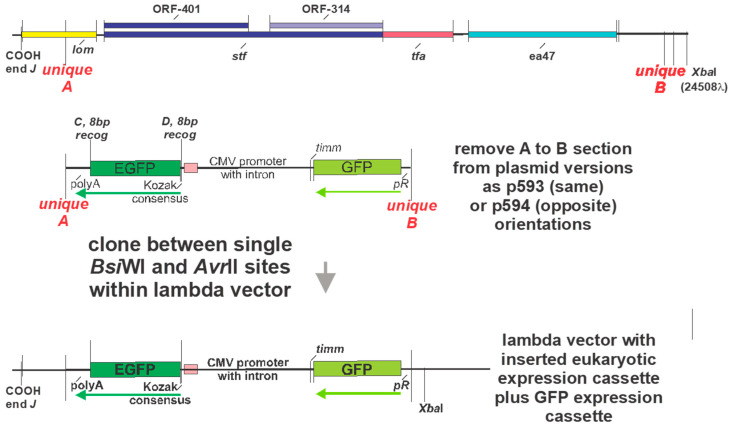
Alternative cloning technique to introduce a eukaryotic expression cassette from plasmid into a vector phage genome by substituting for the *lom-ea47* region on phage genome (straddled by unique single restriction endonuclease sites *BsiWI* (unique A) and *AvrII* (unique B) with the eukaryotic expression cassette present in plasmids as p593 and p594 that lack the purple and orange recombination regions shown for plasmids in Figure 8A and Figure 9A. This reaction requires in vitro λ packaging of the purified, then ligated phage arms with the *BsiWI* to *AvrII* stuffer DNA band, purified from one of the plasmid constructs.

In providing that the pSJH vectors could enable substitutional (gene exchange) recombination (Figure 8A), i.e., replacing a dispensable portion of the λ genome with the eukaryotic expression cassette, copies of DNA from λ genome were introduced into the plasmids straddling the eukaryotic expression cassette. These included segments representing λ bases 18,968–19,328 and 23,937–24,310, color-coded as purple and orange regions, respectively (Figure 8). Their presence in possible vector phage to make LDNAP was examined by PCR (Figure 8B). Surprisingly, only four of the nine potential vector phages carried both regions, and did not include the λ*imm434cI#5* phage that we had used extensively in developing LDP (Figure 5 and Figure 7). An example showing recombinational uptake of a pSJH plasmid eukaryotic expression cassette straddled by the purple and orange DNA homologies into LDNAP vector is shown (Figure 9A), where the LDNAP phage plaques incorporating the eukaryotic expression cassette for EGFP can be detected by hybridization by all of the purified LDNAP plaques (Figure 9B). A diagram of the cloning sites A and B for moving a eukaryotic expression cassette from one of the plasmids into a vector phage containing purple and orange homologies into a vector phage to make an LDNAP is shown (Figure 10). Versions of the pSJH plasmids include GFP gene (replacing antibiotic resistance as a selectable marker and placed under control of the lambda *pR* promoter) within the eukaryotic expression cassette, that is to be moved into a λ vector. This figure shows that λ gene *lom* is substituted (either by recombination or cloning) when the cassette is moved into λ. LDNAP vectors made from essentially wild-type λ phage will tolerate ~6044 bp (12.5%λ) substitutions. As noted, these LDNAP vector recombinants can be modified to make L2DP, i.e., display-coated LDNAP for targeting cell receptors.

### 3.6. Progress and Remaining Work to Exploit LDNAP as Vaccine Agents

Several recent reviews have examined the potential utility of using phage particles as vaccine delivery vehicles [114,115,116,117,118,119,120,121] and the cellular immune responses. We previously demonstrated (a) that LDP displaying fused gpD-CAP epitopes [74] of the Porcine Circovirus 2 (PCV2) produced both neutralizing IgG antibody (in an in vitro assay) and a T-cell-mediated cellular immune response to intradermal vaccination [75] and (b) that LDP displaying gpD-pRp disease-specific prion protein epitopes patterned to combat chronic wasting disease in cervids and formulated as a mucosal vaccine was taken up in Peyer’s patches and the draining mesenteric lymph nodes in claves, and resulted in both mucosal IgA and IgG immune responses [73]. In each of these published vaccination assays the LDP proved immunogenic, but a lot of work is required to assess whether this vaccination strategy, which does not require adjuvant, stimulates a protective immune response.

Further work is needed to prepare and evaluate sheathed DNA vaccines using the LDNAP cloning or recombination technology described herein in order to update the original studies pursued by March and collaborators. Many problems with this technology require a solution; each of them is linked to being able to follow the fate and immune responses of LDNAP administered to and expressed within dendritic cells. What is also important is learning if protective immune responses are evoked in vaccinated animals. Phage lambda includes two genes, *lom* and *bor*, that require consideration relative to using the phage as an approved human vaccine agent. The *bor* gene produce has the potential to confer serum resistance to an infected host cell [122] and the *lom* gene product [113] is involved in *E. coli* K12 bacterial adhesion to human buccal epithelial cells. It is unclear but unlikely either of these proteins are present on the phage capsid and may only influence the bacterial host cells by supporting them to become resistant to a mammalian immune response. The cloning or recombination procedures (Figure 9 and Figure 10) for inserting a eukaryotic cassette within lambda will remove *lom*.

### 3.7. Use of LDP as Antimicrobials to Antimicrobial-Resistant Bacteria, or a Nontargeted Version of Phage Therapy?

We have shown that human and porcine cathelicidins LL37 and PR39 linked to the COOH-terminal end of gpD are highly toxic when expressed *within E. coli*, and similar results were observed for reduced versions of selected human α- and β-defensins. Coated phage particles comprising LDP with gpD-fused cathelicidins or defensins are generated simply by infecting cells capable of expressing a gpD fusion protein below the lethal expression temperatures, i.e., below 41–42 °C. Alternatively, LDP can arise in expression cells where the gpD fusion is only toxigenic when administered to the outer surface of cells. We have found that *D-*defective λ phages are complemented efficiently (50–90^+^%) at infection temperatures of 37–39 °C by cultures of all the toxigenic gpD fusions with cathelicidins and defensins. Clearly, the cathelicidins and defensins evaluated retained biological activity when fused at the COOH end of gpD. LDP lysates produced via using a *D-*defective infecting phage can be employed to generate LDP coated with pathogen-sensitive gpD fusions. Preparation of antimicrobial agents made from LDP will require screening for display peptides that exhibit external cellular toxicity. In such a display system, none of the produced LDP would encode the toxic gpD fusion gene, nullifying horizontal gene transfer.

## 4. Summary, Conclusions, and Other Considerations

### 4.1. Summary and Conclusions

**Figure 11:**
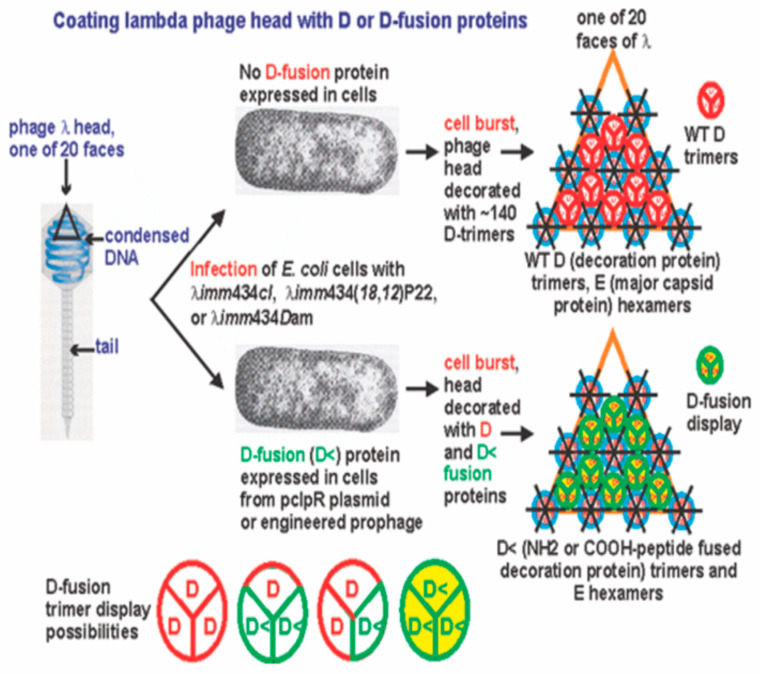
Summary of gpD and gpD< fusion as trimer additions to lambda capsid head.

It is easily possible to produce fully coated LDP, or to produce partially coated LDP without any complex bacteriophage genetic engineering, making the system available to all, as outlined in Figure 11. These LDPs can represent vehicles with utility for stimulating an immune response to the displayed epitope(s), or could serve as antimicrobial agents when displaying an antimicrobial peptide, or be agents for any of the many possible uses suggested by Gupta et al. [25].

Infecting cells expressing gpD fusion proteins from a surrogate plasmid with a lambda phage defective in gene D (i.e., a phage incapable of expressing wild-type gpD) can result in the formation of phage capsids heads having full replacement of gpD by the gpD fusion protein. These fusion particle heads may limit the addition of phage tail structures.

Multiple single-epitope LDP vaccine reagents can be generated in a single infection lysate.

A system was introduced whereby either intracellular phage–plasmid substitution recombination, or by cloning can generate sheathed DNA vaccine particles, termed LDNAP that have the advantage of high-level eukaryotic expression cassette without incorporating plasmid resistance elements or other genes.

### 4.2. Other Considerations

#### 4.2.1. Tailless Phage Particles

Careful examination of Figure 1A (three left lanes) reveals that the level of gpV is much reduced, when compared with that from a gpD competent phage (right-hand lane, Figure 1A, and lanes 1, 2 Figure 7). In earlier studies purifying phage particles coated with gpD-CAP derived from segments of the Porcine Circovirus 2 capsid, we observed the near absence of phage tails in electron micrographs of the banded phage particles [74]. At the time of this earlier publication, we suspected that the absence of tails in the EM photos showing mainly the purified capsid heads was caused by display the Circovirus capsid epitopes, or that the tails were rapidly lost from capsid heads partially coated with gpD< fusion proteins during pelleting in CsCl. Comparing the gpV bands in Figure 1A with Figure 7A suggests that gpD fusions to the lambda capsid head may interfere with the addition of pre-formed tails to the head. Perhaps, in addition, perturbing the proper level of gpD availability can negatively influence the binding of stable tails to capsid heads, reducing the expected level of a gpV band on a protein gel of the disrupted phage particles. In producing a codon-optimized version of gpD (Dcoe), fifty-five of its codons were optimized [74], suggesting that the natural gene had evolved a reduced level of expression. Some possibilities for the perturbation of gpD level include reduction via poorly efficient gpDam123 amber suppression, or an increased level of gpD (Dcoe) when expressed from surrogate plasmid. The absence of phage tails on purified lambda capsid heads coated with a foreign antigen is a preferred situation relative to the preparation of SEV phage particles, since the antigenic load represented by the tail structure is reduced.

#### 4.2.2. gpD-gpD< Trimer Heterogeneity and the Importance of a Spacer

Figure 11 illustrates the possibility that gpD and gpD< additions onto the gpE preformed prohead scaffold may generate heterogeneous trimers with different combinations of gpD and gpD<, rather than homogeneous gpD or gpD< trimers. This requires analysis beyond the scope of this work to resolve. As noted previously, structural studies on gpD trimers revealed that both the NH_2_ and COOH terminal ends of gpD are buried underneath the trimer additions to the head [45]. Presumably, the gene fusions that continue from a short spacer enable the fused epitope(s) to extend from underneath the gpD trimers to the surface of the head.

#### 4.2.3. Phage Particles as Vaccines

Phage vaccines do not require an adjuvant, are orally or nasally admissible, and can be genetically inactivated without denaturing displayed antigens, thus preventing their transmission in nature. Lambda can be stabilized without refrigeration and is stable over a wide pH range [107]. The latter characteristics are ideal for veterinary vaccines and vaccines in developing countries. Phages can act as natural adjuvants of both the cellular and humoral arms of the immune system, triggering immune response that can activate dendritic cells [123,124]. Injecting mice intravenously with 2 × 10^11^ phage particles gave rise to significant levels of interferon [125] which were not due to endotoxin contamination. The serum from these mice bled 18 h after injection was inhibitory to vesicular stomatitis virus in mouse L-cells. Neither the phage DNA nor phage particles without DNA produced IFN levels as high as whole phage particles. It is likely that a combination of foreign DNA containing CpG motifs and the repeating peptide motif of the phage coat can contribute to nonspecific immune stimulation [105,108]. About 711 CpG sequences are within the first 10,000 bases (48,502 bp total not scanned) on one strand, plus 106 CpGpCpG sequences and 20 CpGpCpGpC sequences per same strand of λ genome. Whether any of these sequence motifs are in the correct context to be immunostimulatory or immunoinhibitory remains unanswered at present. Of note, the uptake of lambda DNA into a eukaryotic cell nucleus has been examined [126].

#### 4.2.4. LDNAP and L2DP

It is noted that the LDNAP phage can be propagated in bacterial cells expressing a gpD fusion protein, forming L2DP vaccine particles that could target ligands on antigen presenting cells, increasing the specificity of the sheathed DNA vaccine particle. One idea would be to coat the LDNAP with antigens that will bind dendritic cells, making what I have termed L2DP. Numerous receptors have been identified to be expressed on dendritic cells including mannose receptor (MR), DC-SIGN, scavenger receptor (SR), DEC-205, and toll-like receptors, as described in depth [127]. Lambda DNA vaccines have been prepared against different proteins [105,106,107,108,110,111]. The utility of DNA vaccines has been considered [108,128,129,130].

#### 4.2.5. Improving on Genetic System for PD

The suggestion in Figure 11 is to use phage *λimm434(18,12)*P22 for infections of transformed cells with ColE1-type plasmids expressing gpD< fusion proteins when preparing LDP and SEV. It would be even better to add the *Dam*123 mutation or introduce a deletion into *D* as described in plasmid p613* [72] into this phage. The rationale for substituting phage lambda replication initiation genes *P* and *O* with parallel genes from phage P22 is that each of the plasmids described in Table 1 have ColE1 replication origins. We have determined [72] that ColE1 plasmid replication establishment and maintenance within *E. coli* cells is extremely sensitive to gpP, and this effect (rapid plasmid loss) is not exhibited when the gene *P* is substituted by the P22 replication initiation genes. This would provide for longer gpD< fusion expression when preparing LDP by infection of plasmid-transformed cells with lambda vector phage.

## Figures and Tables

**Table 1:** *E. coli* K12 and bacteriophage λ strains and plasmids utilized.

***E. coli* Strains**	**Relevant Genotype**	**Lab #, Source**
594	Sup° cells; F^−^ *lac-3350 gal 2 galT22 rpsl-179* IN(*rrnD-rrnE*)*1*	B10 [78]; Bachman [79]
W3350 (W3350A)	Sup° F^−^ *lac-3350 galK2 galT22* IN(*rrnD-rrnE*)*1*	B12 [76,80]
TC600	*thr1 leuB6 fhuA21 lacY1 glnV44 el4^−^ glpR200 thi1 supE*	B8 [79]
C3026H	See Methods 2.1	B413
W3350 *recD recF*	*recD1903::*Tn*10 recF400::*Tn*5*	B337, made Hayes lab
**Phages**		
λ*imm434cI#5*	*imm434 cI*	5, 957; [81]
λ*imm434cI*[Ts]sus^+^	*imm434 cI*[Ts]	7, B. Goldstein/W. Dove
*imm434cI b2*	*imm434* with large b2 deletion	286, W. Szybalski
λ*imm434cI*DS	*imm434 cI*	425 [82]
λ*imm434cI*	*imm434 cI*	524 W. Szybalski
λ*imm434*(*18,12*)P22	recombinant [81] with λ*cI857*(*18,12*)P22 [82]	684
λWT*nin5*	recombinant prepared [83]	699
φ80*imm434*	recombinant, Hayes lab	74
λ*cI857 Dam123*	from MMS168, L. Thomason, NCI	1026
λ*imm434 Dam123*	from MMS179, L. Thomason, NCI	1027
λ*imm434cI#5 Dam123*	recombinant (λ*cI857 Dam123x* λ*imm434cI#5*)	isolates A2a,b;G1a,b
**Plasmid Construct**	**Construct Description**	**Construction #; Internal Reference ^a^**
pMZS3F	594 transformant of Mammalian expression vector, (6309 bp) [77] received from J.F. Greenblatt, University of Toronto (which contains several parts derived from pCI-neo, Promega)	p452
pC1-neo	594 transformant of Promega, mammalian expression vector	p457 T:4
XL-1 Blue[pGFPuv]	594 transformant of Clontech, GenBank Acc. #U62636 with “green fluorescence” gene (GFP herein)	p461
pGL14.11(LUC 2P)]	594 transformant of Promega, promoter-defective vector	p487 T:4
pEGFP-c1	594 transformant GenBank #U55763; modified 5bp ∆ in MCS Xho1-Hind 111, Hind111 site retained	p490 T:4
pCI-neo(LUC2P)	594 transformant, 1696bp LUC2P PCR frag. Cloned between XhoI/NotI in pC1-neo, 7104 bp	p497 T:4
pCI-neo-EGFP	594 transformant, PCR EGPF from pEGFP-c1, and cloned between XhoI/NotI in pcI-neo, 6152 bp	p499
pcIpR-timm	=pcIpR[GOI]-timm, backbone described, sequence in [73], constructs covered by Canadian and USA patents	p456
pcIpR-Dcoe-timm ^b,c^	=pcIpR-BamHI-D-BsiWI-ClaI-timm; plasmid was modified from plasmid with same name described in [73] by addition of *BsiWI* cloning site between *timm* and *ClaI* site to avoid having to prepare plasmid DNA in *dam*-defective *E. coli* strains, 4930 bp	p613 F:1,2,4,T3
pGFPuv Vector	pUC backbone, GenBank #U62636	
pcIpR-D-GFP-timm	6627 bp[73]	p470 T:2
pcIpR-M-T-GFP-timm	has the NH_2_-terminal amino acids M and T from D sequence, followed by AGT-AAA-GGA sequence from 739 bp GFP gene in Clonetech pUC vector pGFPuv. See reference [73], 5310 bp	p471
p-cIpR-His-Dcoe-timm	=pcIpR-BamHI-His-D-BsiWI-ClaI-timm, sequence His tag with first two amino acids of Dcoe = *Atgcatcatcaccatcaccacggcagtggtcag*atgact, 4963 bp	p614 F:1,5
pcIpR-Dcoe-YML-timm	=Dcoe-GGSGAP_(spacer)_-GYMLGSAMSRP, 4981 bp	p674 F:1,2,4,5
pcIpR-YML-Dcoe-timm	MGYMLGSAMSRP-GSGQ_(spacer)_-Dcoe, 4978 bp	p676 F:2
pcIpR-Dcoe-CAP-timm ^d,e^	Dcoe-GGSGA_(spacer)_-[CAP:65-87(PSWAVDMMRFNINDFLPPGGGSN)]-AAY-[CAP:113-146(QGDRGVGSSAVILDDNFVTKATALTYDPYVNYSS)]-AAY-[CAP: 158-183 (SRYFTPKPVLDSTIDYFQPNNKRNQL)]-AAY-[CAP:194-207: (DHVGLGTAFENSIY)], 5256 bp; CAP = capsid protein of Porcine Circovirus 2	p458 F:3
pcIpR-D-5E-Ver2-timm ^e^	=pcIpR-Dcoe-BVDV2-E2-Ver2 [84] 1-110 gpDcoe-GGSGAP_(spacer)_-[E2:47-56(EGKDLKILKT)] [E2:61-88(KRYLVAVHERALSTSAEFMQIS DGTIAP)]-AAY_(spacer)_-[E2:111-121(KGKFNASLLNG)]-AAY_(spacer)_- [E2:309-372(RDRYFQQYMLKGEWQYWFDLDSVDHHKDYF SEFIIIAVVALLGGKYVLLLLITYTILFG)]; BVDV2 E2 = capsid protein of Bovine Viral Diarrhea Virus 2; BDVD2 codons optimized for *E. coli*.	p521 F:5,7
pcIpR-D-5E-Ver3-timm ^e^	gpD-BVDV2-E2-Ver3 [84] = 1-110 gpD-GGSGAP_(spacer)_-[E2:47-56(EGKDL KILKT)][E2:61-78(KRYLVAVHERALSTSAEF)]-AAY_(spacer)_-[E2:111-121 (KGKFNASLLNG)]-AAY_(spacer)_-[E2:146-160(LDTTVVRTYRRTTPF] -AAY_(spacer)_-[E2:309-372(RDRYFQQYMLKGEWQYWFDLDSVDH HKDYFSEFIIIAVVALLGGKYVLLLLITYTILFG)]; BDVD2 codons optimized for *E. coli*	p522 F:5,7
pcIpR-Dcoe-RBM496-506-timm ^e^	gpD-RBD#3 = Dcoe-GGSGAP_(spacer)_-GFQPTNGVGYQ; RBM = epitope within receptor-binding domain of SARS-CoV-2 capsid spike protein with Cov2 codons optimized for *E. coli*	p768 F:5
pcIpR-Dcoe-RBM482-494-timm ^e^	gpD-RBD#2 = Dcoe-GGSGAP_(spacer)_-GVEGFNCYFPLQS; RBM = epitope within receptor-binding domain of SARS-CoV-2 capsid spike protein with Cov2 codons optimized for *E. coli*	p771 F:5
pcIpR-Dcoe-RBM443-456-timm ^e^	gpD-RBD#1 = Dcoe-GGSGAP_(spacer)_-SKVGGNYNYLYRLF; RBM = epitope within receptor-binding domain of SARS-CoV-2 capsid spike protein with Cov2 codons optimized for *E. coli*	p770 F:5
pcIpR-Dcoe-HβD3-timm ^e^	Dcoe-GGSGAP_(spacer)_-IINTLQKYYCRVRGGRCAVLSCLPKEEQIGKC STRGRKCCRRKK	p616 T:3
pcIpR-Dcoe-DEFβ126[Δ32]-timm ^e^	Dcoe-GGSGAP_(spacer)_-KSLLFTLAVFMLLAQLVSGNWYVKK CLNDVGICKKKCKPEEMHVKNGWAMCGKQRDCCVPADRRANY PVFCVQTKTTR	p618 T:3
pcIpR-LL37-His-TAGZ-Dcoe-TEV-LL37 ^e^	MHHHHHHGSGQ(ΔM)-Dcoe-GGSGAP_(spacer)_-AGSENLYFQA FALLGDFFRKSKEKIGKEFKRIVQRIKDFLRNLVPRTES	p619 T:3
pcIpR-Dcoe-PR39-timm ^e^	Dcoe-G G S G A P_(spacer)_-RRRPRPPYLPRPRPPPFFPPRLPPRIPPGFPPRF PPRFPGKR	p625 T:3
pcIpR-Dcoe-LL37-timm ^e^	Dcoe-GGSGAP_(spacer)_-FALLGDFFRKSKEKIGKEFKRIVQRIKD FLRNLVPRTES	p627 T:3
pcIpR-Dcoe-HD5 ^e^	Dcoe- GGSGAP_(spacer)_-AGSENLYFQARATCYCRTGRCATRESLSGV CEISGRLYRLCCR	p628 T3
pSJH-D’[EGFP]	726 bp EGFP removed by PCR from pEGFP-c1 and inserted between *AscI-NotI* into pSJH-D’, 4950 bp	p500 T:3
pSJH-D’-LUC2P	1696 bp PCR frag. LUC2P cloned Asc1-Not1 into pSJH-D’, 5954 bp	p498 T:3
pSJH-D’-EGFP] ΔROP	1696 bp PCR frag. LUC2P cloned Asc1-Not1 into pSJH-D’ Derived from p500 = 594[pSJH-D’-EGFP] ΔROP with [23937-24310λ, insert- Avr11/EcoR1][pR-MT-GFP-timm][EGFP-1] [18958-19328λ insert Pci1/BsiW1,](5556bp)	p578 F:9A
pSJH-D’-EGFP] ΔROP	Derived from p500 with [18968-19325λ insert- EcR1/Avr1][pR-MT-GFP-timm] [LUC2P][23937-24310λ insert BsiW1/Pci1, ΔROP], 6492bp	p579 F:9A
p593 ^f^	[23937-24310λ insert- Avr11/EcoR1][pR-MT-GFP-timm] [EGFP-1][18958-19328λ insert Pci1/BsiW1, ΔROP], 5556 bp. pR-MT-GFP transcription from inserted cassette is in same orientation as lambda phage transcription; plasmid made from p500	p593 F:9A
p594 ^f^	[18968-19325]λ insert-EcR1/Avr11][pR-MT-GFP-timm] [EGFP-1][23937-24310λ insert BsiW1/Pci1, ΔROP]. pR-MT-GFP transcription from inserted cassette is in opposite direction as lambda phage transcription; plasmid made from p500	p594 F:9A
p595 ^f^	[23937-24310λ insert- Avr11/EcoR1][pR-MT-GFP-timm][EGFP-2][18958-19328λ insert Pci1/BsiW1, ΔROP]. pR-MT-GFP transcription from inserted cassette is in same orientation as lambda phage transcription; plasmid made from P490 as modified	p595 F:9A
p596 ^f^	[18968-19325λ insert-EcR1/Avr11][pR-MT-GFP-timm][EGFP-2][23937-24310λ insert BsiW1/Pci1, ΔROP]. pR-MT-GFP transcription from cassette is in opposite direction as lambda phage transcription; plasmid made from p490 as modified]	p596 F:9A

^a^ F: figure #, T: table #; ^b^ Dcoe is same amino acid sequence as for *D* wild type, but with 55 codons optimized for usage in *E. coli*: MTSKETFTHYQPQGNSDPAHTATAPGGLSAKAPAMTPLMLDTSSRKLVAWDGTTDGAAVGILAVAADQTSTT LPEAASDETKKRTAFAGTAISIV; ^c^ all pcIpR constructs have an ochre chain termination codon following inserted GOI; ^d^ 66 CAP codons optimized for usage in *E. coli*; all non-*E. coli* codons were optimized for *E. coli* expression; ^e^ Eukaryotic codons optimized for *E. coli*; ^f^ NOTE: Plasmids p593 and p594 (both derivatives of p578), and p595 and p596 (both derivatives of p579) each have an added prokaryotic pR-M-T-GFP-timm cassette from p471 driven by the rightward lambda *pR* promoter as a reporter for directly identifying recombinant phage clones; however, we did not possess an instrument with the sensitivity for detecting GFP expression within a single plaque.

**Table 2:** Temperature dependence of thermally inducible intra-cellular gpD-GFP fusion expression from plasmid pcIpR-D-GFPuv-timm [flurorescence units × 10^−3^].

Induction Time (min)	Culture Up-Shift from 30 °C ^a^		
	37 °C	39 °C	42 °C
0	0	0	3.2
60	0.8	23.2	518.5
120	7.3	30.0	721.2
180	23.9	136.9	1039.5

^a^ The triplicate average for 594 cells (no plasmid) growing at 30 °C and up-shifted to 37, 39, or 42 °C for the indicated times was subtracted from the triplicate average for upshifted 594[pcIpR-D-GFP-timm] whole cells (for more detail refer to [85] and Methods 2.10). gpD-GFP is a fusion protein generated by fusing the DNA sequence for green fluorescence protein to DNA sequence for λ capsid decoration protein gpD. Limited expression from the *pR* promoter was previously observed at 35 to 37 °C where the gene for the highly toxic P protein was cloned downstream of *pR* in pcIpR-P-timm ([86]). The ability of the Ts CI857 repressor encoded by the plasmid to reduce transcription from *pR* begins to be lost at about 35 °C (see results for complementation by cells with p613, Table 1) and the repressor is fully inactive at 42 °C.

**Table 3:** Relative toxicity of gpD-fused α- and β-defensins and cathelicidins.

Host Strain ^a^	Expression Plasmid	Polypeptide Fused to 6 Residue Spacer to COOH End of gpDcoe	Efficiency of Cell Plating		
			30 °C	37 °C	41–42 °C
594	p613	None	1.0	1.0	1.0
594	p616 ^b^	-HβD3	1.0	0.97	<0.00001
C3026H		44 residue β-defensin β-defensin	1.0	0.91	0.87
594	p618 ^b^	-DEFβ126[Δ 32 residues at COOH]	1.0	0.77	<0.00001
C3026H		79 residues β-defensin	1.0	0.96	<0.00001
594	p628 ^b^	-HD5	1.0	1.0	0.0034
C3026H		43 residue α-defensin	1.0	0.78	0.55
594	p625 ^b^	-PR39	1.0	1.0	<0.0001
C3026H		42 residue pig cathelicidin	1.0	0.55	<0.0001
594	p627	-LL37	1.0	0.95	<0.0001
C3026H		39 residue human cathelicidin	1.0	0.56	<0.0001
594	p619	-LL37 [NH_2_-His-tag 11 residue;	1.0	0.77	<0.0001
C3026H		55 residue:COOH-LL37 cathelicidin	1.0	0.88	0.65

^a^ Strain C3026H is available from New England Biolabs, with reported genotype (see Methods) with null mutations in *DsbC* and *trxB* making it defective in reducing formed disulfide bonds. This strain was found (in this work, as control) to have an inducible amber suppressor (active at 37 and 42 °C but not at 30 °C, which made it impossible to distinguish if an infecting *Dam123* phage was able to plaque on the cells at 37 or 42 °C via complementation by gpD fusion protein expressed from plasmid in C3026H transformed cells, or if plaquing was due to suppression of the *Dam123* phage by the unreported inducible suppressor within strain. (Plasmids are described in Methods, Table 1). The procedure for assessing gpD< expression toxicity by measuring EOP is described in Methods. ^b^ The cysteines available for disulfide bond formation in plasmids p616, p618 and p628 are noted in sequences shown (Table 1).

**Table 4:** Expression of EGFP and Luc2P, plasmid comparison ^a^.

Plasmid (Each 1 µg)	Assay 1	Assay 2	Control
pEGFP-C1	45,486	53,486	17,758
pSJH-D’[EGFP]	139,833	138,174	16,867
pGl14.11-Luc2P	810	830	90
pSJH-D’-[Luc2P]	6,530,850	6,556,440	60
pCI-neo[Luc2P]	7,107,550	7,258,200	80

^a^ Expression of EGFP and Luc2P with fluorescence measured, respectively, at 48 and 72 h post-transfection of Cos7 tissue culture cells, each with one microgram (~2 × 10^11^ copies) of plasmid. The plasmids are described in Methods (Table 1). Considerations for assaying GFP and EGFP were reported [86]. pGl14.11 is a promoterless vector control from Promega. Both the EGFP and luciferase readout of Luc2P expression was done in 96 well plates on VictorV/Perkin Elmer spectrophotometer, measuring fluorescence for one second at eight mm height. Negative control values represent general tissue culture backgound values. All values in relative light units. Note: This was conducted as a blind study (samples A–E) where technician did not know identity of provided plasmid DNA and was only given the volume required for 2 × 10^11^ plasmid DNA copies per sample. The controls for EGFP samples showed high tissue culture background and were not filter-optimized for EGFP mesurement and were done in regular 96 well plates, rather than specialized (black bottom) plates generally recommended for fluorescence/luminescense to avoid cross-talk.

## Data Availability

Data is contained within the article or Supplementary Material. Individual DNA sequence information can be requested from the author.

## Supplementary Materials

The following supporting information can be downloaded at: https://www.mdpi.com/article/10.3390/v17111406/s1, Plasmid Construction/Considerations Example.
