# Supplementary material for: Potential Vaccine or Antimicrobial Reagents: Simple Systems for Producing Lambda Display Particles (LDP) and Sheathed Lambda DNA Vaccine Particles (LDNAP)
Source: Viruses. 2025 Oct 22;17(11):1406. doi: 10.3390/v17111406 (PMC12656833; doi:10.3390/v17111406)
Supplement: Supplementary file 1 [file viruses-17-01406-s001.zip › viruses-3766021-supplementary.pdf]

Supplemental: Plasmid Construction Example:

Plasmid Construction Example/Considerations. Brief summary of eukaryotic expression plasmid constructions, pSJH-A – pSJH-E. These five plasmids were custom designed and constructed for the purpose of preparing LDNAP, *i.e.,* for the *purpose of placing a eukaryotic expression cassette for a vaccine gene into a bacteriophage lambda DNA cloning vector that was optimized for safety/security*. For genomic integration and expression: pSJH-A: INS-CMV-INT-MCS-BGHPA-INS and pSJH-B: INS-CMV-INT-MCS-SV40PA; for gene expression: pSJH-C: CMV-INT-MCS-BGHPA-INS, pSJH-D: CMV-INT-MCS-SV40PA, pSJH-D':CMV-INT-MCS-SV40PA (EcoRI-BsiWIXbaI) (all plasmids reported herein are derived from pSJH-D'), and for expression and replication in nucleus: pSJH-E: CMV-INT-MCS-SV40PA-Φfi-SV40ori. They are used to clone the GOI, which needs to be codon optimized for expression in eukaryotic cells. The GOI is positioned downstream of powerful eukaryotic promoter-intron combination and upstream of eukaryotic polyadenylation signal. In the pSJH plasmids the MCS was designed as AscI-XhoI-NotI-ClaI, where AscI and NotI have rare 8bp target sites, and the ClaI site adjacent to SV40PA does not have an adjacent "C", so that there is no GATC Dam methylation to inhibit ClaI restriction endonuclease. In addition, the restriction sites straddling the eukaryotic cassette are: EcoRI-SstI-[KpnI-Eukaryotic cassette]-BsiWI-XbaI in pSJH-D'(EcoRI-BsiWIXbaI). In all of the pSJH plasmids the bacterial T7 and T3 promoters present in pCI-neo and pMZS3F were genetically removed so that they wouldn't interfere when the vector plasmid is grown in *E. coli*. The MCS was reduced to 30 base pairs. [Example: preparing 4290 bp pSJH-D' (EcoRI-BsiWIXbaI) plasmid: 1) Remove KpnI – MfeI fragment from pRλ*c*I857 (Hayes lab) and retain 2934 bp band. 2) Ligate 2984 bp fragment with ligated PCR fragment (made in several PCR/ligation steps from 1096 and 1365 bp intermediates) representing *Kpn*I-*Bgl*II-CMV-INT-*Ase*I-*Xho*I-*Not*I-*Cla*I-SV40PA-*EcoR*I to make pSJHD [4356 base pairs].~~Step~~ 2~~)~~: Delete T7 promoter [in pMZS3F & pCI-neo] and the T3 promoter and MCS in pCI-neo. 3) Remove bases 1-1417 between *EcoR*I and *Xba*I sites and, substitute designed *EcoR*I --- *BsiW*I-*Xba*I PCR fragment, eliminating 67 base pairs (including 12 restriction sites including *Cla*I) from pSJH-D to make pSJH-D(*EcoR*I-*BsiW*I-*Xba*I) = pSJH-D'.] The plasmids eukaryotic expression cassettes were derived from plasmid pMZS3F (6309 bp) [125] received from J.F. Greenblatt, Univ. of Toronto (which contains parts derived from pCI-neo (Clonetech)). All of the plasmids include an intron (INT) derived from plasmid pCI-neo, that is positioned between the CMV promoter and the MCS in original plasmids. The intron was included because studies have shown that if introns are deleted from a gene, its RNA product is exported much more slowly to the cytoplasm. This suggests that the intron may provide a signal for the attachment of the export apparatus. In all pSJH plasmids the bacterial T7 and T3 promoters present in pCI-neo and pMZS3F were removed so that they would not interfere when the vector plasmid is grown in *E. coli*. As noted, the original MCS was reduced to

30 base pairs. Versions B, D and E of pSJH encode the SV40-PA. Versions A and C have the BGH-PA. In pSJH-A the eukaryotic expression cassette is straddled by "insulator" (INS) sequences, while plasmid pSJH-C has an insulator sequence downstream of the BGH-PA sequence. In version pSJH-E the SV40 origin is incorporated so that the segment can replicate within a eukaryotic cell. pSJH-E may be used to make a more powerful, but less controllable LDNAP.

Example: p593 = pSJH-D'[MTGFP][EGFP]=5556bp, MTGFP transcription in SAME orientation.

RED= Lambda Fragment inserted left of eukaryotic expression cassette: XbaI tcTaga18968 to 19325cctagg AvrII

TEAL removed lambda Or3 – through SalI site to make pR-MT-GFP-timm for insertion in sSJH-D' plasmids with GFP addition

YELLOW WITH GREEN INTRON INSERT: CMV promoter with intron from pCI-neo

Pink overlay = Kozak consensus translation sequence, where last AT is start of EGFP gene

Grey overlay = EGFP gene

Brown = Lambda fragment inserted Right of Eukaryotic expression cassette: *BsiW*I **cgtacg23937 to 24310acatgt *Pci*I**

Gaattctctagacgtaatgtgtgtattgccgttgctgtctttgccgcacttgcggtgacagtcactccggcccgtgcggaagg
tggacatggtacgtttacggtgggctattttcaagtgaaaccgggtacattgccgtcgttgtcgggcggggataccggtgagt
catctgaaagggattaacgtgaagtaccgttatgagctgacggacagtgtgggggtgatggcttccctggggttcgccgcgtcg
aaaaagagcagcacagtgatgaccggggaggatacgtttcactatgaga*gcctgcgtggacgttatgtga*gcgtgatggccg
gaccggttttacaaatcagtaagcaggtcagtgcgtcctaggtatcaccgcaagggataaatatctaacaccgt
gcgtgttgactattttacctctggcggtgataatggttgcatgtactaaggaggatccATGACT
AGTAAAGGAGAAGAACTTTTCACTGGAGTTGTCCCAATTCTTGTTGAATTAGATGGTGATGTTA
ATGGGCACAAATTTTCTGTCAGTGGAGAGGGTGAAGGTGATGCAACATACGGAAAACTTACCCT
TAAATTTATTTGCACTACTGGAAAACTACCTGTTCCATGGCCAACACTTGTCACTACTTTCTCT
TATGGTGTTCAATGCTTTTCCCGTTATCCGGATCATATGAAACGGCATGACTTTTTCAAGAGTG
CCATGCCCGAAGGTTATGTACAGGAACGCACTATATCTTTCAAAGATGACGGGAACTACAAGAC
GCGTGCTGAAGTCAAGTTTGAAGGTGATACCCTTGTTAATCGTATCGAGTTAAAAGGTATTGAT
TTTAAAGAAGATGGAAACATTCTCGGACACAAACTCGAGTACAACTATAACTCACACAATGTAT
ACATCACGGCAGACAAACAAAAGAATGGAATCAAAGCTAACTTCAAAATTCGCCACAACATTGA
AGATGGATCCGTTCAACTAGCAGACCATTATCAACAAAATACTCCAATTGGCGATGGCCCTGTC
CTTTTACCAGACAACCATTACCTGTCGACACAATCTGCCCTTTCGAAAGATCCCAACGAAAAGC
GTGACCACATGGTCCTTCTTGAGTTTGTAACTGCTGCTGGGATTA*CACATGGCATGGATGAGCT*
*CTACAAATA*AtcgatcccggggtcagccccgggttttcttttGAATTcgTCGACggtaccAGAT
CTTCAATATTGGCCATTAGCCATATTATTCATTGGTTATATAGCATAAATCAATATTGGCTATT
GGCCATTGCATACGTTGTATCTATATCATAATATGTACATTTATATTGGCTCATGTCCAATATG
ACCGCCATGTTGGCATTGATTATTGACTAGTTATTAATAGTAATCAATTACGGGGTCATTAGTT
CATAGCCCATATATGGAGTTCCGCGTTACATAACTTACGGTAAATGGCCCGCCTGGCTGACCGC
CCAACGACCCCCGCCCATTGACGTCAATAATGACGTATGTTCCCATAGTAACGCCAATAGGGAC

TTTCCATTGACGTCAATGGGTGGAGTATTTACGGTAAACTGCCCACTTGGCAGTACATCAAGTG
TATCATATGCCAAGTCCGCCCCCTATTGACGTCAATGACGGTAAATGGCCCGCCTGGCATTATG
CCCAGTACATGACCTTACGGGACTTTCCTACTTGGCAGTACATCTACGTATTAGTCATCGCTAT
TACCATGGTGATGCGGTTTTGGCAGTACACCAATGGGCGTGGATAGCGGTTTGACTCACGGGGA
TTTCCAAGTCTCCACCCCATTGACGTCAATGGGAGTTTGTTTTGGCACCAAAATCAACGGGACT
TTCCAAAATGTCGTAACAACTGCGATCGCCCGCCCCGTTGACGCAAATGGGCGGTAGGCGTGTA
CGGTGGGAGGTCTATATAAGCAGAGCTCGTTTAGTGAACCGTCAGATCACTAGAAGCTTTATTG
CGGTAGTTTATCACAGTTAAATTGCTAACGCAGTCAGTGCTTCTGACACAACAGTCTCGAACTT
AAGCTGCAGTGACTCTCTTAAGGTAGCCTTGCAGAAGTTGGTCGTGAGGCACTGGGCAGGTAAG
TATCAAGGTTACAAGACAGGTTTAAGGAGACCAATAGAAACTGGGCTTGTCGAGACAGAGAAGA
CTCTTGCGTTTCTGATAGGCACCTATTGGTCTTACTGACATCCACTTTGCCTTTCTCTCCACAG
GTGTCCACTCCCAGTTCAATTACAGCTCTTggcgcgccactcgaggtcgccaccATGGTGAGCA
AGGGCGAGGAGCTGTTCACCGGGGTGGTGCCCATCCTGGTCGAGCTGGACGGCGACGTAAACGG
CCACAAGTTCAGCGTGTCCGGCGAGGGCGAGGGCGATGCCACCTACGGCAAGCTGACCCTGAAG
TTCATCTGCACCACCGGCAAGCTGCCCGTGCCCTGGCCCACCCTCGTGACCACCCTGACCTACG
GCGTGCAGTGCTTCAGCCGCTACCCCGACCACATGAAGCAGCACGACTTCTTCAAGTCCGCCAT
GCCCGAAGGCTACGTCCAGGAGCGCACCATCTTCTTCAAGGACGACGGCAACTACAAGACCCGC
GCCGAGGTGAAGTTCGAGGGCGACACCCTGGTGAACCGCATCGAGCTGAAGGGCATCGACTTCA
AGGAGGACGGCAACATCCTGGGGCACAAGCTGGAGTACAACTACAACAGCCACAACGTCTATAT
CATGGCCGACAAGCAGAAGAACGGCATCAAGGTGAACTTCAAGATCCGCCACAACATCGAGGAC
GGCAGCGTGCAGCTCGCCGACCACTACCAGCAGAACACCCCCATCGGCGACGGCCCCGTGCTGC
TGCCCGACAACCACTACCTGAGCACCCAGTCCGCCCTGAGCAAAGACCCCAACGAGAAGCGCGA
TCACATGGTCCTGCTGGAGTTCGTGACCGCCGCCGGGATCACTCTCGGCATGGACGAGCTGTAC
AAGTAAgcggccgcatcgatgagcagacatgataagatacattgATGAGTTTGGACAAACCACA
ACTAGAATGCAGTGAAAAAAATGCTTTATTTGTGAAATTTGTGATGCTATTGCTTTATTTGTAA
CCATTATAAGCTGCAATAAACAAGTTAACAACAACAATTGCATTCATTTTATGTTTCAGGTTCA
GGGGGAGATGTGGAGGTTTTTTAAAGCAAGTAAAACCTCTACAAATGTGGTAAAATccgataa
ggcgtacgAggttgcatgctagatgctgatatatttagaggtgataaaattaactgcttaactgtcaatgtaatac
aagttgtttgatcttt*gcaatgattcttatcagaaacc*atatagtaaattagttacacaggaatttttaatattat
tattatcattcattatgtattaaaattagagttgtggcttggctctgctaacacgttgctcataggagatatggtag
agccgcagacacgtcgtatgcaggaacgtgctgcggctggctggtgaacttccgatagtgcgggtgttgaatgattt
ccagttgctaccgatttacatatttttgcatgagagaatttgtaccacctcccaccgaccatctatgactgtaca
tgtGAGCAAAAGGCCAGCAAAAGGCCAGGAACCGTAAAAAGGCCGCGTTGCTGGCGTTTTTCCAT
AGGCTCCGCCCCCCTGACGAGCATCACAAAAATCGACGCTCAAGTCAGAGGTGGCGAAACCCGA
CAGGACTATAAAGATACCAGGCGTTTCCCCCTGGAAGCTCCCTCGTGCGCTCTCCTGTTCCGAC
CCTGCCGCTTACCGGATACCTGTCCGCCTTTCTCCCTTCGGGAAGCGTGGCGCTTTCTCATAGC
TCACGCTGTAGGTATCTCAGTTCGGTGTAGGTCGTTCGCTCCAAGCTGGGCTGTGTGCACGAAC
CCCCCGTTCAGCCCGACCGCTGCGCCTTATCCGGTAACTATCGTCTTGAGTCCAACCCGGTAAG
ACACGACTTATCGCCACTGGCAGCAGCCACTGGTAACAGGATTAGCAGAGCGAGGTATGTAGGC
GGTGCTACAGAGTTCTTGAAGTGGTGGCCTAACTACGGCTACACTAGAAGGACAGTATTTGGTA
TCTGCGCTCTGCTGAAGCCAGTTACCTTCGGAAAAAGAGTTGGTAGCTCTTGATCCGGCAAACA
AACCACCGCTGGTAGCGGTGGTTTTTTTGTTTGCAAGCAGCAGATTACGCGCAGAAAAAAAGGA
TCTCAAGAAGATCCTTTGATCTTTTCTACGGGGTCTGACGCTCAGTGGAACGAAAACTCACGTT
AAGGGATTTTGGTCATGAGATTATCAAAAAGGATCTTCACCTAGATCCTTTTAAATTAAAAATG
AAGTTTTAAATCAATCTAAAGTATATATGAGTAAACTTGGTCTGACAGTTACCAATGCTTAATC
AGTGAGGCACCTATCTCAGCGATCTGTCTATTTCGTTCATCCATAGTTGCCTGACTCCCCGTCG
TGTAGATAACTACGATACGGGAGGGCTTACCATCTGGCCCCAGTGCTGCAATGATACCGCGAGA
CCCACGCTCACCGGCTCCAGATTTATCAGCAATAAACCAGCCAGCCGGAAGGGCCGAGCGCAGA

AGTGGTCCTGCAACTTTATCCGCCTCCATCCAGTCTATTAATTGTTGCCGGGAAGCTAGAGTAA
GTAGTTCGCCAGTTAATAGTTTGCGCAACGTTGTTGCCATTGCTGCAGGCATCGTGGTGTCACG
CTCGTCGTTTGGTATGGCTTCATTCAGCTCCGGTTCCCAACGATCAAGGCGAGTTACATGATCC
CCCATGTTGTGCAAAAAAGCGGTTAGCTCCTTCGGTCCTCCGATCGTTGTCAGAAGTAAGTTGG
CCGCAGTGTTATCACTCATGGTTATGGCAGCACTGCATAATTCTCTTACTGTCATGCCATCCGT
AAGATGCTTTTCTGTGACTGGTGAGTACTCAACCAAGTCATTCTGAGAATAGTGTATGCGGCGA
CCGAGTTGCTCTTGCCCGGCGTCAACACGGGATAATACCGCGCCACATAGCAGAACTTTAAAAG
TGCTCATCATTGGAAAACGTTCTTCGGGGCGAAAACTCTCAAGGATCTTACCGCTGTTGAGATC
CAGTTCGATGTAACCCACTCGTGCACCCAACTGATCTTCAGCATCTTTTACTTTCACCAGCGTT
TCTGGGTGAGCAAAAACAGGAAGGCAAAATGCCGCAAAAAAGGGAATAAGGGCGACACGGAAAT
GTTGAATACTCATACTCTTCCTTTTTCAATATTATTGAAGCATTTATCAGGGTTATTGTCTCAT
GAGCGGATACATATTTGAATGTATTTAGAAAAATAAACAAATAGGGGTTCCGCGCACATTTCCC
CGAAAAGTGCCACCTGACGTCTAAGAAACCATTATTATCATGACATTAACCTATAAAAATAGGC
GTATCACGAGGCCCTTTCGTCTTCAA
